# Antifouling Napyradiomycins from Marine-Derived Actinomycetes *Streptomyces aculeolatus* †

**DOI:** 10.3390/md18010063

**Published:** 2020-01-18

**Authors:** Florbela Pereira, Joana R. Almeida, Marisa Paulino, Inês R. Grilo, Helena Macedo, Isabel Cunha, Rita G. Sobral, Vitor Vasconcelos, Susana P. Gaudêncio

**Affiliations:** 1 LAQV, Chemistry Department, Faculty for Sciences and Technology, NOVA University of Lisbon, 2829-516 Caparica, Portugal; florbela.pereira@fct.unl.pt; 2 CIIMAR/CIMAR—Interdisciplinary Centre of Marine and Environmental Research, University of Porto, Terminal de Cruzeiros do Porto de Leixões, Avenida General Norton de Matos, 4450-208 Matosinhos, Portugal; joana.reis.almeida@gmail.com (J.R.A.); isabel.cunha@ciimar.up.pt (I.C.); vmvascon@fc.up.pt (V.V.); 3 UCIBIO, Chemistry Department, Blue Biotechnology and Biomedicine Lab, Faculty for Sciences and Technology, NOVA University of Lisbon, 2829-516 Caparica, Portugal; m.paulino@campus.fct.unl.pt (M.P.); h.macedo@campus.fct.unl.pt (H.M.); 4 UCIBIO, Life Sciences Department, MOLMICRO of Bacterial Pathogens Lab, Faculty for Sciences and Technology, NOVA University of Lisbon, 2829-516 Caparica, Portugal; inesgrilo@fct.unl.pt (I.R.G.); rgs@fct.unl.pt (R.G.S.); 5 Biology Department, Faculty of Sciences, Porto University, Rua do Campo Alegre, 4069-007 Porto, Portugal

**Keywords:** marine natural products, actinomycetes, biofouling, antifouling, antibiofilm, napyradiomycins, meroterpenoids, hybrid isoprenoids, drug discovery, bioprospection

## Abstract

The undesired attachment of micro and macroorganisms on water-immersed surfaces, known as marine biofouling, results in severe prevention and maintenance costs (billions €/year) for aquaculture, shipping and other industries that rely on coastal and off-shore infrastructures. To date, there are no sustainable, cost-effective and environmentally safe solutions to address this challenging phenomenon. Therefore, we investigated the antifouling activity of napyradiomycin derivatives that were isolated from actinomycetes from ocean sediments collected off the Madeira Archipelago. Our results revealed that napyradiomycins inhibited ≥80% of the marine biofilm-forming bacteria assayed, as well as the settlement of *Mytilus galloprovincialis* larvae (EC_50_ < 5 µg/ml and LC_50_/EC_50_ >15), without viability impairment. In silico prediction of toxicity end points are of the same order of magnitude of standard approved drugs and biocides. Altogether, napyradiomycins disclosed bioactivity against marine micro and macrofouling organisms, and non-toxic effects towards the studied species, displaying potential to be used in the development of antifouling products.

## 1. Introduction

Marine biofouling is the undesired accumulation of micro and macroorganisms on submerged surfaces, including bacteria, algae, larvae, and adults of various phyla, and their by-products, in a dynamic process that begins immediately after water-submersion and takes hours to months to develop [1]. Biofouling formation is divided into four distinct phases: soon after the physical adherence of macromolecules, the process becomes biological, designated as the microfouling phase, in which a bacterial biofilm is responsible for the establishment of an appropriate surface for the subsequent macrofouling organisms to settle, first as spores and larvae which then develop into adults [2,3]. 

Marine biofouling creates risk to several industries such as aquaculture, power plants, and shipping, amongst others [4,5]. Settlement on the vessel´s hull damages the rudder and propulsion systems [4,6], and leads to an increasing drag of up to 60%, requiring up to 40% higher fuel consumption, in addition to increased CO_2_ and SO_2_ emissions [7]. Moreover, hull biofouling and ballast water transfer are the main causes for the introduction and spread of nonindigenous marine species into ecosystems worldwide leading to environmental imbalances [8,9,10,11,12]. Antifouling (AF) methods are estimated to save the shipping industry around €60 billion/year in fuel [4]. The most effective AF coatings contain biocides, such as tributyltin (TBT) and tributyltin oxide (TBTO), which were proven to be harmful to non-target organisms and the environment [13]. In fact, the International Maritime Organization banned TBT from ship surfaces in 2008, sparking the demand for new generations of nontoxic or environmentally benign AF solutions [14,15,16].

In the last years, several reviews reported studies on natural products (NP) isolated from marine organisms with AF activity [17,18,19,20,21,22,23,24,25]. The quest for AF agents from marine sources started with 2-furanone bromine derivatives extracted from red algae that were reported to prevent fouling [26]. Oroidin, a bromopyrrole alkaloid with AF activity isolated from sponges, inspired the design of 50-synthetic analogs [27,28]. An interesting approach to create AF “living” paints was developed [29] using marine bacteria that were directly encapsulated into polyurethane coatings [30] and hydrogels [31]. Regarding actinomycetes as AF sources, lobocompactol, a diterpene from *Streptomyces cinnabarinus*, was active against the macroalgae *Ulva pertusa*, the diatom *Navicula annexa,* and the bacterium *Pseudomonas aeruginosa* [32,33]. The 6-benzyl and 6-isobutyl 2,5-diketopiperazine derivatives from *S. praecox* were also active against *U. pertusa* and *N. annexa* [34]. 2,5-Diketopiperazines from *S. fungicidicus* and a branched-chain fatty acid, 12-methyltetradecanoid acid, from *Streptomyces* sp. inhibited the barnacle *Balanus amphitrite* and the polychaeta *Hydroides elegans* larval attachment, respectively [35,36,37]. Quercetin, a flavonoid obtained from *S. fradiae* revealed activity against the cyanobacteria *Anabena sp.* and *Nostoc sp.*, and mussel *Perna indica* larvae [38]. To the best of our knowledge, ivermectin, a chemically modified form of avermectin, a macrolide isolated from *S. avermitilis*, commonly used to treat parasitic worms and as insecticide, is the only marketed AF agent obtained from actinomycetes, although terrestrial, which is used in paints for macrofouling inhibition.

We focused on bioprospecting marine-derived actinomycetes as producers of biofouling inhibitors for the potential development of marine-derived sustainable antifouling products. Napyradiomycins are a class of hybrid isoprenoids and/or meroterpenoids known for their antimicrobial and anticancer activities [39,40]. The napyradiomycins reported herein were isolated from a marine-derived actinomycete collection obtained from ocean sediments collected off the Madeira Archipelago [41]. The napyradiomycin molecular network with antibiofilm statistical bioactivity prediction was reported in a recent study by our group [42]. Here, we describe the capacity of napyradiomycins isolated from *S. aculeolatus* to inhibit micro and macrofouling species, and evaluate their ecotoxicity using an in silico approach. Targeting the primary attachment phases of the fouling process would allow preventing accumulation of other marine species. We recently patented the use of napyradiomycin derivatives for marine antifouling paints and coatings [43].

## 2. Results and Discussion

### 2.1. Napyradiomycin Derivatives Description

**Figure 1:**
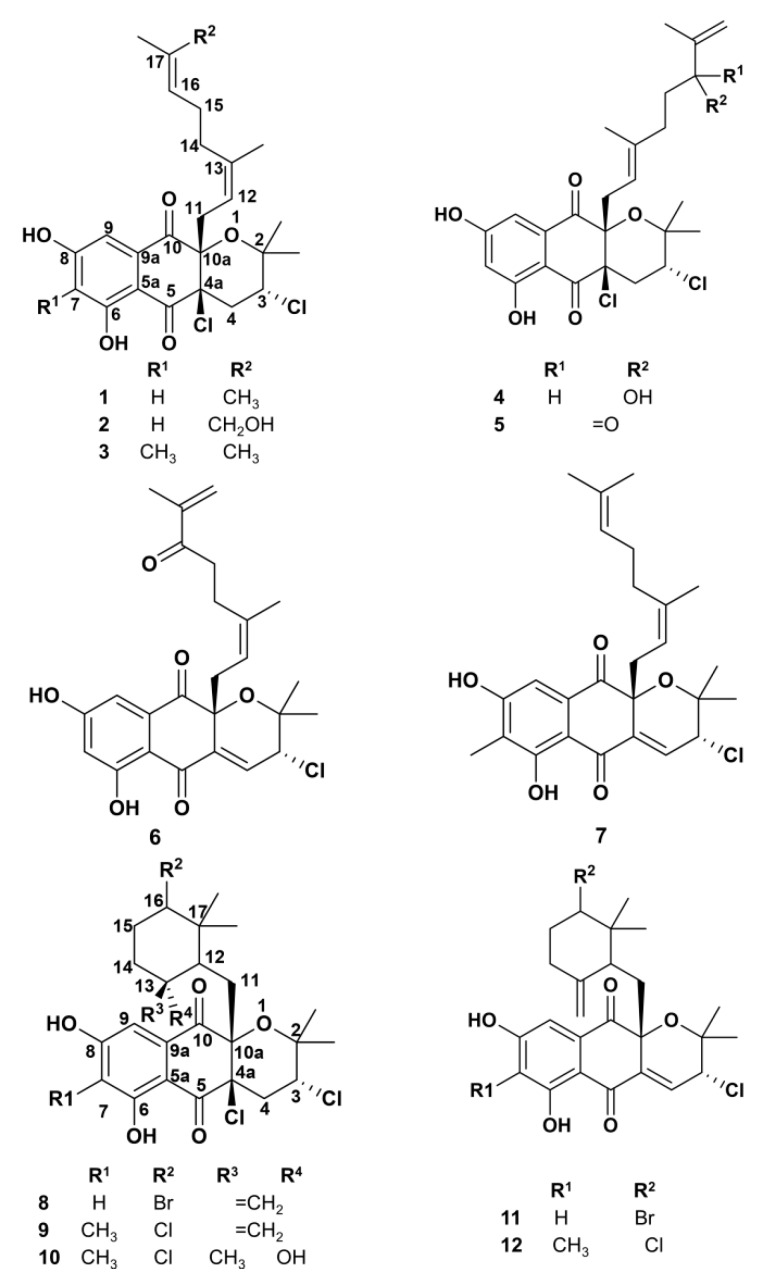
Chemical structures of napyradiomycins isolated from marine-derived *S. aculeolatus* strains PTM-029 (**3**), (**7**), (**9**), (**10**), and (**12**) and PTM-420 (**1**), (**2**), (**4**–**6**), (**8**), and (**11**).

Ethyl acetate (EtOAc) extracts of *Streptomyces aculeolatus* PTM-029 and PTM-420 [41,42] were subjected to micro and macro antifouling bioassay-directed fractionation and isolation, first by silica flash chromatography and subsequently by C_18_ reversed-phase HPLC to yield nine samples comprising twelve napyradiomycin derivatives (**1**–**12**) (Figure 1). The structures of all compounds were established by HR-MS and interpretation of NMR spectroscopic data, especially 2D NMR (i.e., COSY, HSQC, HMBC, TOCSY experiments), and by comparing the data with those previously reported for napyradiomycins [39,44,45,46,47,48,49]. All isolated napyradiomycins (**1**–**12**) were previously reported and their structural characterization is described in the Supplementary Materials. Napyradiomycins SF2415B3 (**3**), 4-dehydro-4a-dechloro- napyradiomycin SF2415B3 (**7**), A80915A (**9**), A80915C (**10**), 4-dehydro-4a-dechloro- napyradiomycin A80915A (**12**) and A1 (**1**), 18-hydroxynapyradiomycin A1 (**2**), A2 (**4**), 16-oxonapyradiomycin A2 (**5**), 4-dehydro-4a-dechloro-16-oxonapyradiomycin A2 (**6**), B3 (**8**), 4-dehydro-4a-dechloro-napyradiomycin B3 (**11**) were isolated from strains PTM-029 and PTM-420, respectively. Interestingly, there was a marked difference in these two sets of napyradiomycins: all the napyradiomycins isolated from PTM-029 (**3**), (**7**), (**9**), (**10**), and (**12**) have a methyl group in the core structure at position 7, while the ones obtained from strain PTM-420 (**1**), (**2**), (**4**–**6**), (**8**), and (**11**) have a hydrogen atom in that position (Figure 1). 

Despite some of the napyradiomycins were isolated as a mixture (**3** and **7**), (**5** and **6**), (**8** and **11**), and (**9** and **12**) no further purification efforts were performed, since antifouling agents are commonly used as mixture of compounds. For example, ivermectin antifouling product is commercialized as a mixture of two homologous compounds, ~80% of ivermectin B1a, with an ethyl group at position C-26 and ~20% of ivermectin B1b, with a methyl group at C-26 [50].

### 2.2. Assessment of Napyradiomycin Derivatives Micro and Macrofouling Inhibitory Activity 

The antimicrofouling activity of napyradiomycins (**1**–**12**) was evaluated by testing their inhibitory activity on bacterial propagation and bacterial biofilm formation. Five species of marine bacteria, which are biofilm prolific and described as fouling effectors, including dominant primary colonizers of submerged surfaces [51], were chosen as models for our bioactivity assays, namely *Marinobacter hydrocarbonoclasticus* (DSM 8798), *Cobetia marina* (DSM 4741)*, Micrococcus luteus* (DSM 20030, ATCC 4698), *Pseudooceanicola batsensis* (DSM 15984) and *Phaeobacter inhibens* (DSM 17395) [52,53,54,55,56,57,58]. 

#### 2.2.1. Antibacterial Activity

As a first screening approach, liquid cultures of *M. hydrocarbonoclasticus,* and *C. marina* in a 96-well format were analyzed for bacterial growth inhibition by percent decrease in OD_600_ after incubation with napyradiomycins (**1**–**12**) at 31.25 µg/mL. 

No PTM-029 napyradiomycins had activity against *M. hydrocarbonoclasticus* or *C. marina*, while PTM-420 napyradiomycins (**1**) and (**8** and **11**) revealed antibacterial activity against *C. marina.* Therefore, PTM-420 napyradiomycins were further tested against three other marine bacterial species, *M. luteus, P. batsensis,* and *P. inhibens*. 

**Figure 2:**
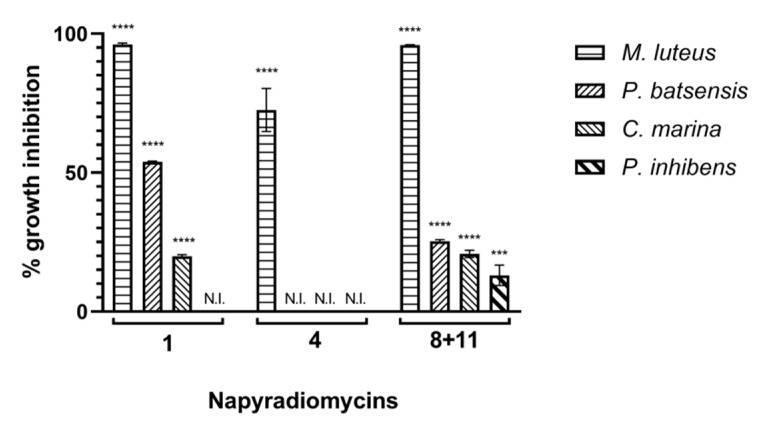
Growth inhibition assay performed for napyradiomycins (**1**–**12**), at a concentration of 31.25 µg/mL. After incubation for 18 h, bacterial growth was assayed by measuring OD_600nm_. The growth of *M. hydrocarbonoclasticus* was not inhibited by any of the napyradiomycins tested at this concentration, while (**2**), (**3** and **7**), (**5** and **6**), (**9** and **12**), (**10**), and (**12**) did not inhibit growth of any of the bacteria tested. Percentage of growth inhibition refers to the percentage of growth that was inhibited in the presence of napyradiomycins, when compared to growth with only DMSO. Shown are the average results of three replicates and error bars represent the standard error of the mean (SEM). N.I.—not inhibited; results were statistically significant (**** *p* < 0.0001, *** *p* < 0.001, Dunnett’s test).

Napyradiomycin (**1**) inhibited the growth of *M. luteus, P. batsensis,* and *C. marina* (96.1 ± 0.5%, 53.9 ± 0.3%, and 19.8 ± 0.6%, respectively), while (**8** and **11**) inhibited the growth of the same three species in addition to *P. inhibens* (95.9 ± 0.1%, 25.3 ± 0.6%, 20.8 ± 1.3%, and 13.0 ± 3.7%, respectively). Napyradiomycin (**4**) only inhibited the growth of *M. luteus* (72.5 ± 7.7%), at this concentration (Figure 2). The remaining napyradiomycins (**2**) and (**5** and **6**) had no effect on bacterial growth of these marine bacterial species, at a concentration of 31.25 µg/mL. 

**Figure 3:**
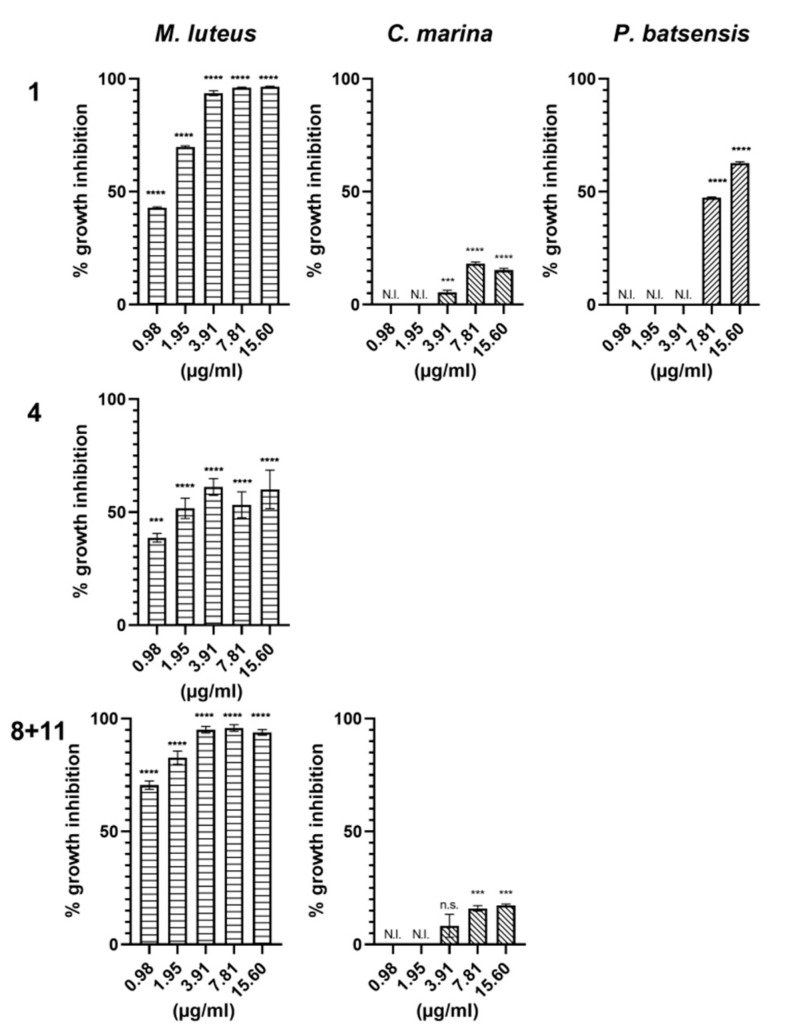
Bacterial growth inhibition of *M. luteus*, *C. marina,* and *P. batsensis* by napyradiomycins (**1**), (**4**), and (**8** and **11**). Napyradiomycins were added to the growth medium of the different bacteria and after 18 h incubation, bacterial growth was assayed by measuring OD_600nm_. Percentage of growth inhibition refers to the percentage of growth that was inhibited in the presence of the napyradiomycins, when compared to growth with only DMSO. Shown are the average results of three replicates and error bars represent the standard error of the mean (SEM). N.I. not inhibited; results were statistically significant (**** *p* < 0.0001, *** *p* < 0.001, n.s. *p* > 0.05, Dunnett’s test).

Napyradiomycins (**1**), (**4**) and (**8** and **11**) were further assayed at lower concentrations (serial 2-fold dilutions, 15.60 µg/mL to 0.98 µg/mL), against the bacterial species for which they showed antibacterial activity at a concentration of 31.25 µg/mL (Figure 3 and Table 1). 

Napyradiomycin (**1**) drastically inhibited (>90%) the growth of *M. luteus* at a concentration of 3.91 µg/mL. At the lowest tested concentration, 0.98 µg/mL, (**1**) significantly inhibited the growth of *M. luteus* (42.9 ± 0.4%). For *C. marina,* (**1**) was active at higher concentrations, albeit with a lower growth inhibition percentage (5.5 ± 0.9% for 3.91 µg/mL; 18.2 ± 0.7% for 7.81 µg/mL, and 15.3 ± 0.7% for 15.60 µg/mL). The growth of *P. batsensis* was also significantly inhibited by this napyradiomycin at the concentration of 7.81 µg/mL (47.4 ± 0.3%) and 15.60 µg/mL (62.7 ± 0.6%).

Napyradiomycin (**4**) was able to significantly inhibit approximately 50% of the growth of *M. luteus* at all tested concentrations.

Napyradiomycins (**8** and **11**) drastically inhibited (>90%) the growth of *M. luteus* at a concentration of 3.91 µg/mL. At the lowest concentration, 0.98 µg/mL, (**8** and **11**) still inhibited bacterial growth significantly (70.5 ± 1.9%). The growth of *C. marina was* also significantly inhibited by (**8** and **11**) at concentrations of 7.81 µg/mL (16.0 ± 1.2%), and 15.60 µg/mL (17.4 ± 0.5%). Although (**8** and **11**) inhibited the bacterial growth of *P. inhibens* and *P. batsensis* at a concentration of 31.25 µg/mL their growth was not inhibited at the lower tested concentrations (15.60 µg/mL to 0.98 µg/mL).

CuSO_4_ (5 μM), a potent antifouling agent used in antifouling paints, was used as reference. The growth of *P. inhibens*, *P. batsensis*, and *M. hydrocarbonoclasticus* was inhibited by CuSO_4_ (66.2 ± 5.6%, 44.8 ± 6.3%, and 35.8 ± 0.8%). *M. luteus* and *C. marina* growth was not inhibited. 

#### 2.2.2. Antibiofilm Activity

**Figure 4:**
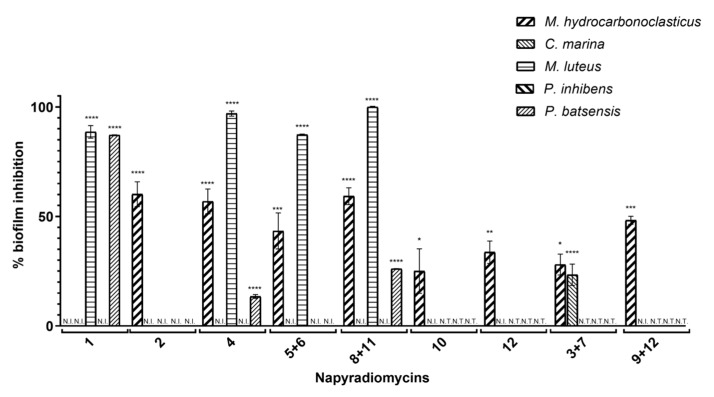
Biofilm inhibition assay performed using napyradiomycins (**1**–**12**), at a concentration of 31.25 µg/mL. After incubation for 18 h, planktonic cells were washed, and biofilm was stained using crystal violet and measured at OD_600nm_. The biofilm formation of *P. inhibens* was not inhibited by any of the napyradiomycins tested at this concentration. Percentage of biofilm inhibition refers to the percentage of biofilm that was inhibited in the presence of the napyradiomycins, when compared to biofilm formation with only DMSO. Shown are the average results of three replicates and error bars represent the standard error of the mean (SEM). N.I.—not inhibited; N.T.—not tested, results were statistically significant (**** *p* < 0.0001, *** *p* < 0.001, ** *p* < 0.01, * *p* < 0.05; Dunnett’s test).

In a 96-well plate, biofilm grown cultures of *M. hydrocarbonoclasticus* and *C. marina* were incubated with napyradiomycins (**1**–**12**) at a concentration of 31.25 µg/mL. After discarding the media, biofilm inhibition was determined from OD_600_ measurements of crystal violet stained cells resuspended from the plate bottom with acetic acid. All napyradiomycins extracted from the actinomycete strain PTM-029 (**3** and **7**), (**9** and **12**), (**10**), and (**12**) showed significant biofilm inhibition towards *M. hydrocarbonoclasticus* that ranged from 25.1 ± 10.2% for (**10**) to 48.2 ± 1.9% for (**9** and **12**). Additionally, napyradiomycins (**3** and **7**) also showed significant biofilm inhibition towards *C. marina* (23.4 ± 4.9%) (Figure 4 and Table 2).

All PTM-420 napyradiomycins, except (**1**) showed significant antibiofilm activity against *M. hydrocarbonoclasticus* (**4**, 56.9 ± 5.7%; **8** and **11**, 59.3 ± 3.8%; **5** and **6,** 43.4 ± 8.2%; **2**, 60.2 ± 5.7%).

As for the bacterial growth inhibition assays, compounds isolated from PTM-420 were tested against three other marine bacterial species, *M. hydrocarbonoclasticus, C. marina, M. luteus, P. batsensis,* and *P. inhibens* (Figure 4 and Table 2). 

Napyradiomycin (**4**) showed significant antibiofilm activity against *M. luteus* and *P. batsensis* (97.0 ± 1.2%, and 13.4 ± 0.8%, respectively). Likewise, (**8** and **11**) also showed significant antibiofilm activity against *M. luteus* and *P. batsensis* (100.0 ± 0.3% and 26.2 ± 0.0%, respectively). Napyradiomycin (**1)** had high antibiofilm activity against *M. luteus* and *P. batsensis* (88.6 ± 2.9% and 87.2 ± 0.1%, respectively), while (**5** and **6**) inhibited the biofilm formation of *M. luteus* (87.3 ± 0.3%) (Figure 4 and Table 2).

**Figure 5:**
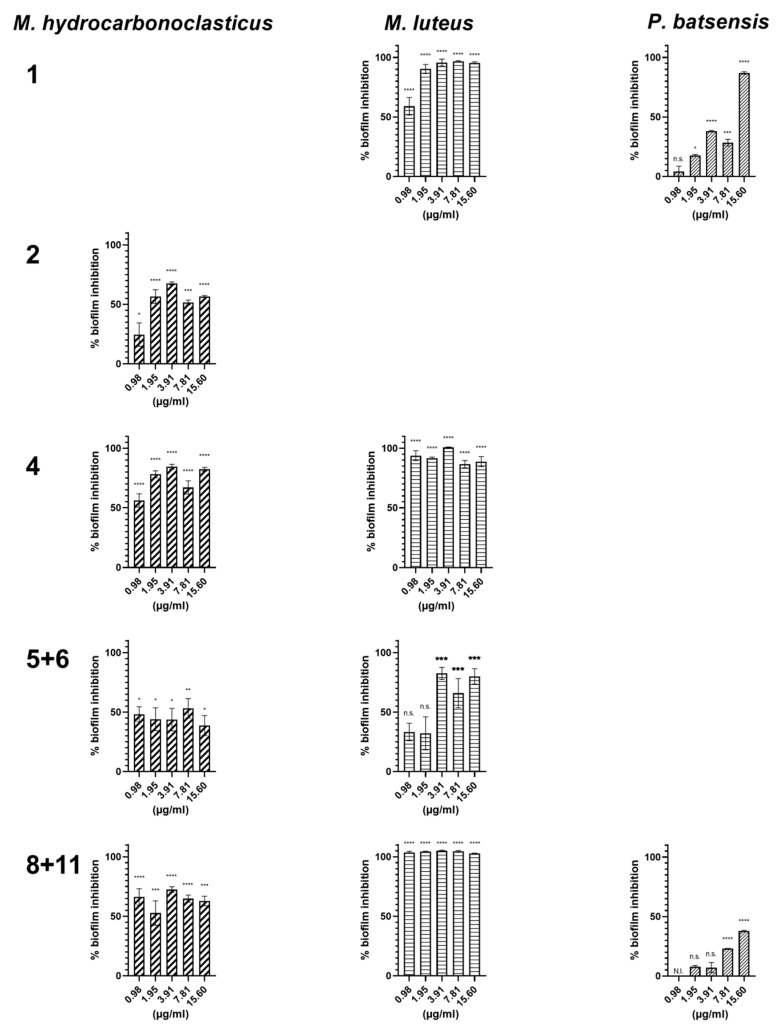
Inhibition of biofilm formation of *M. hydrocarbonoclasticus, M. luteus,* and *P. batsensis* by napyradiomycins (**1**), (**2**), (**4**), (**5** and **6**), and (**8** and **11**). Napyradiomycins were added to the biofilm growth medium of the different bacteria and after 18 h incubation planktonic cells were washed, and biofilm was stained using crystal violet and measured at OD_600nm_. Percentage of biofilm inhibition refers to the percentage of biofilm that was inhibited in the presence of the napyradiomycins, when compared to biofilm formation with only DMSO. Shown are the average results of three replicates and error bars represent the standard error of the mean (SEM). N.I.—not inhibited; results were statistically significant (**** *p* < 0.0001, *** *p* < 0.001, ** *p* < 0.01, * *p* < 0.05, n.s. *p* > 0.05, Dunnett’s test).

PTM-420 napyradiomycins, were assayed at lower concentrations (serial 2-fold dilutions, 15.60 µg/mL to 0.98 µg/mL), against the bacterial species for which they showed antibiofilm activity at a concentration of 31.25 µg/mL (Table 3 and Figure 5). 

Napyradiomycins (**8** and **11**) completely abolished (100%) biofilm formation of *M. luteus* at all tested concentrations. Biofilm formation by this species was nearly completely inhibited (>90%) by (**1**) at concentrations higher than 1.95 µg/mL, and at the lowest concentration of 0.98 µg/mL inhibition was of 59.0 ± 7.4%. At this concentration, biofilm formation was also nearly eliminated by (**4**) (>90%). Napyradiomycins (**5** and **6**) significantly inhibited the biofilm formation (~80%) of *M. luteus*, at a concentration of 3.91 µg/mL and higher. Biofilm formation of *M. hydrocarbonoclasticus* was inhibited by (**2**), (**4**), (**5** and **6**), and (**8** and **11**) by at least 50% at all tested concentrations and 80% in the case of (**4**). 

*P. batsensis* biofilm formation was inhibited by (**8** and **11**) at a concentration of 1.95 µg/mL and higher. Napyradiomycin (**1**) also efficiently inhibited biofilm formation of *P. batsensis* (86.9 ± 1.0%) at a concentration of 15.60 µg/mL, and was moderately active at lower concentrations (Table 3).

The most promising compounds for antimicrofouling are those that inhibit biofilm formation without killing the bacteria. Compounds (**4**), (**5** and **6**), (**8** and **11**), and (**3** and **7**) have potential in this respect, as all inhibit biofilm formation of at least two of the marine bacterial species assayed without inhibiting their growth at the same concentration.

Specifically, (**4**) inhibited the growth of only *M. luteus* but was able to inhibit biofilm formation of *M. luteus* and *M. hydrocarbonoclasticus* (Figure 4 and Table 2). In addition, at the lowest tested concentration (0.98 µg/mL), the antibacterial activity against *M. luteus* was 38.7 ± 7.7%, while biofilm inhibition was over 90%. Therefore, (**4**) showed antimicrofouling effectiveness at low concentrations. 

Napyradiomycins (**5** and **6**) showed no antibacterial activity, but high biofilm inhibition of *M. luteus* and *M. hydrocarbonoclasticus* (e.g., for a concentration of 3.91 µg/mL, biofilm formation of *M. luteus* was inhibited by 82.5 ± 5.1%, while biofilm formation by *M. hydrocarbonoclasticus* was inhibited by 43.8 ± 9.4%).

Napyradiomycins (**8** and **11**) showed inhibitory activity of biofilm formation and a variable degree of antibacterial activity against three of the five tested marine bacteria. For *M. hydrocarbonoclasticus*, (**8** and **11**) did not inhibit growth but significantly inhibited biofilm formation (~60%) for all tested concentration. For *M. luteus*, growth inhibition and complete biofilm abolishment (100%) was observed for all tested concentrations, however for the lowest concentration, growth inhibition was lower (70.5 ± 1.9%), indicating selective antibiofilm activity. For *P. batsensis*, growth inhibition (25.3 ± 0.6%) was observed only for the highest tested concentration (31.25 µg/mL), but significant inhibition of biofilm formation was observed at lower concentrations (37.8 ± 0.7% at a concentration of 15.60 µg/mL), with no detected antibacterial activity).

Finally, napyradiomycins (**3** and **7**) significantly inhibited (>20%) biofilm formation of *M. luteus* and *C. marina*, at a concentration of 31.25 µg/mL, while not showing any antibacterial effect on these marine bacterial species.

The antibiofilm activity of these napyradiomycins is not dependent on their antibacterial activity, which means that these compounds could be used as AF agents that will not contribute to antibiotic/biocide resistance.

The biofilm formation of *P. inhibens* and *P. batsensis* was inhibited by CuSO_4_ (12.9 ± 6.4% and 41.4 ± 0.9%). *M. luteus, M. hydrocarbonoclasticus,* and *C. marina* biofilm formation was not inhibited. 

### 2.3. Antifouling Evaluation against Mytilus Galloprovincialis Larval Settlement

Napyradiomycins (**1**–**12**) were screened against *M. galloprovincialis* plantigrade larval settlement to assess antimacrofouling activity. Most reports use tropical species and only a few involve temperate or cold-water species [59,60], we recognized the need to use European fouling species able to grow in temperate climate in bioassays. 

The compounds revealed EC_50_ values ranging from 0.10 to 6.34 µg/mL (Table 4). 

All compounds revealed a good level of effectiveness, which is defined by an EC_50_ value < 25 µg/mL [61]. The most effective napyradiomycins, showing an EC_50_ below 1 µg/mL were (**1**) (EC_50_ 0.66 µg/mL), (**8** and **11**) (EC_50_ = 0.73 µg/mL), (**9** and **12**) (EC_50_ = 0.45 µg/mL), (**10**) (EC_50_ = 0.10 µg/mL), and (**12**) (EC_50_ = 0.95 µg/mL) (Table 4). Interestingly, the EC_50_ of (**10**) was better than the commercial agent ivermectin (EC_50_ = 0.4 µg/mL value against *M. edulis*) [62]. 

Regarding toxicity, none of the tested compounds caused mortality of *M. galloprovincialis* larvae at the highest tested concentration (12 µg/mL). Thus, LC_50_ values were considered higher than 12 µg/mL. The EC_50_ and LC_50_ values were used to calculate the therapeutic ratios (LC_50_/EC_50_). To meet the standard requirement for efficacy level of natural antifouling agents the US Navy program established a cut-off above 15 for the therapeutic ratio [21]. Therefore, the most promising antifouling agents towards *M. galloprovincialis* larvae are napyradiomycins (**1**) (LC_50_/EC_50_ = 18.3), (**8** and **11**) (LC_50_/EC_50_ = 16.5), (**9** and **12**) (LC_50_/EC_50_ = 26.6), and (**10**) (LC_50_/EC_50_ = 117.3) (Table 4). 

### 2.4. Napyradiomycins in Silico Ecotoxicity Evaluation

The ecotoxicity of diverse pharmaceuticals, biocides and chemical compounds have forced regulatory authorities to recommend the application of in silico risk assessment to predict the fate of these molecules and their potential ecological and indirect human health effects. Using the Toxicity Estimation Software Tool (T.E.S.T.) [63], napyradiomycins (**1**–**12**) were evaluated for potential ecotoxicity, the prediction results are in Table 5. 

In accordance with the European Union Directive 2001/59/EC and the Regulation on the Classification, Labelling and Packaging of Substances and Mixtures (CLP) 1272/2008, the in silico TEST results classified compounds **1**–**12**, with the danger symbol N (“dangerous for the environment”), risk phrase 50 (“very toxic to aquatic organisms” to “toxic to aquatic organisms”), acute toxicity estimate (ATE) category 4 (“practically non-toxic and not an irritant”), and as developmental toxicants with low bioaccumulation factor and mutagenicity negative [64,65,66,67].

In comparison, predictions of toxicity were performed for seven approved drugs: bimatoprost (**S1**), a topical medication used for controlling the progression of glaucoma or ocular hypertension; alfuzosin (**S2**), a nonselective alpha-1 adrenergic antagonist used in the therapy of benign prostatic hypertrophy; lovastatin (**S3**), a fungal metabolite isolated from cultures of *Aspergillus terreus* and a potent anticholesteremic agent; antimycin A (**S4**), an antibiotic produced by *Streptomyces* sp.; oxethazaine (**S5**), an anesthetic; calcipotriene (**S6**), a synthetic derivative of calcitriol or Vitamin D used for the treatment of moderate plaque psoriasis in adults; and latanoprost (**S7**), a prostaglandin F2alpha analogue and a prostanoid selective FP receptor agonist with an ocular hypertensive effect, (Supplementary Table S1), two antifouling agents (ivermectin B1b (**S8**) and ivermectin B1a (**S9**), (Supplementary Table S2), and arsenic and copper substances used in marine paints (Supplementary Table S3). 

The predicted values related with environmental toxicity for (**1**–**12**) (Table 5) are in the same order of magnitude and have the same classification than those obtained for the Prestwick approved drugs (**S1**–**S7**) (Supplementary Table S1) and the two antifouling approved biocides **S8**, **S9** (Supplementary Table S2). Except for ATE, in which our napyradiomycins showed lower toxicity than **S1**–**S9** (category ≤ 4). 

Comparing the toxicity predictions for (**1**–**12**) (Table 5) with the values for copper (Supplementary Table S3), we may predict that these MNP are less toxic than copper, which is widely used in antifouling paints and coatings [64,65]. Arsenic showed lower aquatic toxicity values than (**1**–**12**) but higher acute toxicity. Overall, the in silico results suggest napyradiomycins as a suitable model to test for Naval Sea Systems Command (NAVSEA) standards and proceed in the antifouling coatings development roadmap (http://www.nstcenter.biz/navy-product-approval-process/navy-community-coatings-roadmap/, accessed on 6th January 2020).

### 2.5. SAR Analysis

To date, 61 napyradiomycin derivatives have been discovered and elucidated [42]. In general, the chemical structure of the napyradiomycins consists of a semi-napthoquinone core, a prenyl unit attached at C-4a that is cyclized to form a tetrahydropyran ring in all the napyradiomycins reported here (Figure 1), and a monoterpenoid substituent attached at C-10a. The variation of the chemical structure of napyradiomycins is mainly due from the monoterpenoid subunit (10-carbon), which can be linear (as the napyradiomycins **1**–**7**) or cyclized to a 6-membered ring (as napyradiomycins **8**–**12**). As mentioned above, napyradiomycins from the strains PTM-029 and PTM-420 differ by a methyl group at position 7 instead of hydrogen, respectively (Figure 1). Overall, the antifouling activities of (**1**–**12**) ranked in descending order are summarized as: (**1**) > (**8** and **11**) > (**4**) for antibacterial activity, (**4**) > (**5** and **6**) > (**8** and **11**) > (**3** and **7**) for antimicrofouling, and (**10**) > (**9** and **12**) > (**1**) > (**8** and **11**) > (**12**) > (**3** and **7**) for antimacrofouling activity. Interestingly, all the napyradiomycins with antibacterial activity and the three napyradiomycins with highest antimicrofouling activity were isolated from the strain PTM-420 with a hydrogen atom at position 7. Conversely, the methyl containing napyradiomycins from PTM-029 generally had higher antimacrofouling activity.

Based on the biosynthetic scheme for napyradiomycins reported by Moore and co-workers [68], (**1**), (**2**), (**4**–**6**), (**8**), (**11**) lack the methylation of flaviolin by the methyltransferase NapB5. Similar behavior occurs with the 10-carbon monoterpenoid subunit, which is linear in almost all of the napyradiomycins with more potent antibacterial and antimicrofouling activities, with the exception of napyradiomycins (**8** and **11**), and cyclized to a 6-membered ring in most of the most active antimacrofouling napyradiomycins, except for napyradiomycins (**1**) and (**3** and **7**). Therefore, our antifouling results (micro and macro) suggest a correlation with this biosynthetic feature. Napyradiomycins (**8** and **11**) stand out as having the ability to inhibit both micro and macrofouling, this feature appears to be related with the presence of a bromine substitute at C-16 (Figure 1). MNP bromide derivatives seem to play an important role as antifouling agents, as 2-furanone bromine derivatives [26] and bromopyrrole alkaloids from oroidin family [27,28].

## 3. Materials and Methods

### 3.1. General Experimental Procedures

The optical rotations were measured using a Bellingham+Stanley (Berlin, Germany), model ADP410 Polarimeter with a 5 dm cell. UV/VIS spectra were recorded using an Ultraspect 3100 Pro Amersham Bioscienses spectrophotometer (Champaign, IL, USA) with a path length of 1 cm, and IR spectra were recorded on a Perkin Elmer Spectrum Two FT-IR Spectrometer (Santa Clara, CA, USA). ^1^H and 2D NMR spectral data were recorded at 400 or 600 MHz in CDCl_3_ containing Me_4_Si as internal standard on Bruker Advance and Bruker BioSpin spectrometers respectively (Ettlingen, Germany). ^13^C NMR spectra were acquired at 100 MHz on a Bruker Advance spectrometer. High resolution ESI-TOF mass spectra were obtained through acquired services using an Agilent 6230 Accurate-Mass TOFMS spectrometer (Santa Clara, CA, USA) by the mass spectrometry facility at the Department of Chemistry and Biochemistry at the University of California, San Diego, La Jolla, CA. Low-resolution LC/MS data were measured through acquired services at MARINOVA, CIIMAR, Portugal, using a Thermo Finnigan Surveyor HPLC System (Thermo Fisher Scientific, Needham, MA, USA), coupled with Mass Spectrometry LCQ Fleet™ Ion Trap Mass Spectrometer (Thermo Fisher Scientific, Needham, MA, USA), with reversed-phase C_18_ column (Phenomenex Luna, 100 mm × 1.0 mm, 5 µm), ACN:H_2_O 10–100% gradient, with 0.1% formic acid, at a flow rate of 0.7 mL/min. 

### 3.2. Collection and Isolation of Marine-Derived Actinomycetes

Sediment samples were collected in June 2012 off shore of the Madeira Archipelago of Portugal. Strain PTM-029 was isolated from samples collect near Madeira Island at 728 m using a dredge, while PTM-420 was isolated from samples collected from Desertas Island at 15 m on SCUBA. The sediments were inoculated using a heat-shock method: wet sediment (c. 0.5 g) was diluted with 2 mL of sterile seawater (SSW). After mixing, the diluted samples were allowed to settle for few minutes, heated to 55 °C for 6 min. 50 µL of the top layer was spread on an agar plate, with seawater based medium SWA (18 g agar per L) with the antifungal cycloheximide (100 µl/L). 

Inoculated Petri dishes were incubated at RT (c. 25–28 °C) and monitored periodically over 6 months for actinomycete growth. The PTM-029 and PTM-420 colonies were successively transferred onto new seawater based A1 medium (10 g starch, 4 g yeast extract, 2 g peptone per L) until the attaining of pure strain. PTM-029 and PTM-420 were grown in liquid culture (without agar) and cryopreserved in 10% (v/v) glycerol at −80 °C.

### 3.3. Phylogenetic Analysis of Strains PTM-029 and PTM-420

Strains PTM-029 and PTM-420 were cultured in 4mL of A1 medium, with agitation (200 rpm) at 25 °C for 7 days. Genomic DNA was isolated using the Wizard^®^ Genomic DNA Purification Kit (Promega, Madison, WI, USA) protocol for Gram positive bacteria. The manufacturer recommendations were followed, though with longer incubation periods of the lytic enzyme (i.e., lysozyme) and the RNase solution to obtain sufficient amounts of genomic DNA. The 16S rRNA gene was amplified using the primers 27F (5′-AGAGTTTGATCCTGGCTCAG-3′) and 1492R (5′-TACGGCTACCTTGTTACGACTT-3′) [41,69] and purified using SureClean PCR cleanup kit (BioLine, London, UK), using the protocol provided by the manufacturer. Purified PCR reactions were cycle- sequenced with the primers listed above at STABVIDA, Lda (www.stabvida.net), using ABI BigDye^®^ Terminator v3.1 Cycle Sequencing Kit (Needham, MA, USA). Purified products were run on an ABI PRISM^®^ 3730xl Genetic Analyzer (Needham, MA, USA) and sequence traces were edited using Sequencing Analysis 5.3.1 from Applied Biosystems™ (Needham, MA, USA). The sequence was compared to the GenBank database by the blastn algorithm. 

PTM-029 and PTM-420 sequences have been deposited in GenBank under accession numbers KP869059 and KP869064 respectively, available at www.ncbi.nlm.nih.gov/genbank. 

### 3.4. Growth Conditions and Crude Extract Production

The actinomycetes (strains PTM-029 and PTM-420) were grown in 20 Erlenmeyer flasks with 2L capacity, each containing 1 L of seawater based A1 medium with agitation (200 rpm) at 30 °C. After seven days of incubation, the culture was extracted thrice with half volume of EtOAc and evaporated to dryness in vacuum to yield ~1.0 g of crude extracts. 

### 3.5. Isolation of Napyradiomycins

The PTM-029 (~1.0 g) and PTM-420 crude extracts (~1.0 g) were fractionated by silica flash chromatography, eluted with step gradients of isooctane/EtOAc followed by EtOAc/MeOH. A mixture of five napyradiomycins from PTM-029 and seven from PTM-420 eluted with the 8:2 and 6:4 fractions of isooctane/EtOAc, respectively, and further isolated by reversed phase HPLC (Phenomenex Luna, 250 mm × 4.6 mm, 5 µm, 100 Å, 1.5 mL/min, UV 210, 250 and 360 nm) using a gradient solvent system from 70% to 100% CH_3_CN in water (0.1% TFA) over 90 min to yield napyradiomycins (**1**, 12.86 mg), (**2**, 5.04 mg), (**3** and **7**, 6.80 mg), (**4**, 11.36 mg), (**5** and **6**, 1.67 mg), (**8** and **11**, 5.21 mg), (**9** and **12**, 7.40 mg), and (**10**, 13.20 mg) as orange oils. The data for structural characterization is described in the Supplementary Materials.

### 3.6. Antimicrofouling Evaluation

#### 3.6.1. Bacterial Growth Conditions

Five marine bacterial species were used as models to assess antimicrofouling activity. The biofilm-forming marine bacteria *Marinobacter hydrocarbonoclasticus* DSM 8798 (ATCC 49840), *Cobetia marina* DSM 4741, *Phaeobacter inhibens* DSM 17,395, and *Pseusooceanicola batsensis* DSM 15,984 were obtained from DSMZ (Leibniz Institute DSMZ—German collection of Microorganisms and Cell Cultures) and *Micrococcus luteus* [55]. Cultures were routinely grown in liquid marine broth (Carl Roth GmbH, Karlsruhe, Germany) with agitation (180 rpm) or on agar-supplemented marine broth, at 28 °C (*M. hydrocarbonoclasticus* and *C. marina*) or 30 °C (*P. inhibens* and *P. batsensis*). *M. luteus* was maintained in Brain Heart Infusion broth (BHI, Becton Dickinson, GmbH, Heidelberg, Germany) with agitation (180 rpm) or on agar-supplemented BHI, at 37 °C.

#### 3.6.2. Antibacterial Activity Evaluation Assays

The antibacterial activity of the napyradiomycins (**1**–**12**) was assessed in 96-well polystyrene flat bottom microplates (Nunclon Delta Surface, Thermo Scientific, Roskilde, Denmark) following previously reported procedures [42]. For initial screening, bacterial overnight cultures were diluted to an optical density (OD_600nm_) of 0.2 and incubated statically at 28 °C (*M. hydrocarbonoclasticus, C. marina*), 30 °C (*P. inhibens, P. batsensis*) or 37 °C (*M. luteus*) in a 96-well microplate in the presence or absence of 31.25 µg/mL of the napyradiomycins, solubilized in DMSO. After 24 h (*M. hydrocarbonoclasticus, C. marina*) or 48 h (*P. batsensis, P. inhibens, M. luteus*) incubation, the OD_600nm_ was determined (Molecular Devices, Spectra Max 190). The napyradiomycins which showed antibacterial activity at a concentration of 31.25 µg/mL were then tested at lower concentrations (2-fold serial dilutions: 15.60, 7.81, 3.91, 1.95, and 0.98 µg/mL), and the protocol was repeated as described above. The percentage of growth inhibition was calculated as the amount of growth relative to that of the bacterial species without added compounds (with the same amount of DMSO added). CuSO_4_ (5 μM), a potent antifouling agent used in antifouling paints, was used as reference.

All assays were performed in triplicate, and results are representative of the average and standard error of the mean (SEM). Statistical analysis was performed in GraphPad Prism 8.0.2 (San Diego, CA, USA), using one-way ANOVA followed by a Dunnett’s multiple comparisons test against the control (species grown with the same amount of DMSO added). 

#### 3.6.3. Antibiofilm Activity Evaluation Assays

The antibiofilm activity of napyradiomycins (**1**–**12**) against the five marine bacterial species was assessed in 96-well polystyrene flat bottom microplates (Nunclon Delta Surface, Thermo Scientific, Roskilde, Denmark) as previously reported [42]. For initial screening, bacterial overnight cultures were diluted to an optical density (OD_600nm_) value of 0.2 and incubated statically at 28 °C (*M. hydrocarbonoclasticus, C. marina*), 30 °C (*P. inhibens, P. batsensis*) or 37 °C (*M. luteus*) in the 96-well microplate in the presence or absence of 31.25 µg/mL of napyradiomycins, solubilized in DMSO. After 24 h (*M. hydrocarbonoclasticus, C. marina*) or 48 h (*P. batsensis, P. inhibens, M. luteus*) incubation, the OD_600nm_ was determined. The planktonic cells and media were discarded, and the wells were washed twice with deionized water. The biofilm was fixed for 1 h at 60 °C and stained with crystal violet 0.06% for 10 min. The dye was discarded, and the wells were again washed twice with deionized water. The stained biofilm was solubilized with 30% acetic acid and the OD_600nm_ was determined. The napyradiomycins which showed antibiofilm activity at a concentration of 31.25 µg/mL were then tested at lower concentrations (2-fold serial dilutions: 15.60, 7.81, 3.91, 1.95, 0.98 µg/mL), and the protocol was repeated as described above. The percentage of biofilm inhibition was calculated as the amount of biofilm relative to that of the bacterial species without added compounds (with the same amount of DMSO added). CuSO_4_ (5 μM), a potent antifouling agent used in antifouling paints, was used as reference.

All assays were performed in triplicate, and results are representative of the average and standard error of the mean (SEM). Statistical analysis was performed in GraphPad Prism 8.0.2, using one-way ANOVA followed by a Dunnett’s multiple comparisons test against the control (species grown with the same amount of DMSO added).

### 3.7. Antimacrofouling Evaluation: Mussel Larvae Mytilus Galloprovincialis Acute Toxicity Assay

Mussel (*M. galloprovincialis*) adhesive larvae (plantigrades) were used to assess in vivo the antifouling activity of napyradiomycins (**1**–**12**) towards macrofouling. Juvenile mussel aggregates were collected at the intertidal rocky shore during low spring tides, at Memória beach, Matosinhos, Portugal (41°13′59″ N; 8°43′28″ W). At the laboratory, immediately before the bioassays, mussel plantigrade larvae were screened and isolated from the juvenile aggregates using a binocular microscope (Olympus SZX2-ILLT, Hamburg, Germany) and washed with filtered seawater to remove organic debris. Only competent plantigrade larvae (those showing foot exploratory behavior) were selected and used in the exposure bioassays following previously validated procedures [70,71]. Plantigrades were exposed in 24-well polystyrene plates for 15 h in the darkness at 18 °C. DMSO was used as solvent for crude extracts, fractions and pure compounds stock and working solutions. Working solutions were prepared by successive dilutions of stock solutions in DMSO and then diluted in filtered seawater to obtain the test solutions. DMSO concentration in test solutions was always 0.1%. Four well replicates were used per condition with five larvae per well. Two negative controls, one with ultra-pure water and the other with DMSO 0.1% were included in all bioassays, as well as a positive control with 5 μM CuSO_4_ (a potent antifouling agent). Anti-settlement bioactivity was determined by the presence/absence of fixed byssal threads produced by each individual larvae for all the conditions tested. Napyradiomycins were tested at 5 µg/mL and those showing anti-settlement activity were tested at higher and lower successive concentrations (12, 6, 3, 1.5, 0.75, and 0.375 µg/mL) for the determination of the semi-maximum response concentrations (EC_50_) that had an anti-settlement effect in mussel larvae. Chi-square Pearsons goodness-of-fit test was applied to data in order to determine EC_50_. Significance was considered at *p* < 0.01 for all analyses, and 95% lower and upper confidence limits [95% LCL; UCL] were presented. Therapeutic ratio (LC_50_/EC_50_) was used to evaluate the effectiveness vs. toxicity of compounds [19,21].

### 3.8. In Silico Environmental Toxicity Assessment 

The in silico toxicity evaluation was done using the Toxicity Estimation Software Tool (T.E.S.T.) [63], https://www.epa.gov/chemical-research/toxicity-estimation-software-tool-test, that was developed to allow users to easily estimate toxicity using a variety of QSAR methodologies. In accordance with the European Union Directive 2001/59/EC and the Regulation on the Classification, Labelling and Packaging of Substances and Mixtures (CLP) 1272/2008, a substance can be classified as “harmful”, “toxic”, and “very toxic” to aquatic organisms depending on the 96-hour LC_50_ for fish (e.g., fathead minnow), 48 hour LC_50_ for daphnids (e.g., *Daphnia magna*), and others assays such as 72-hour IC_50_ for algae or 40 hour IGC_50_ for protozoans (e.g., *Tetrahymena pyriformis*). If IC_50_ or LC_50_ or IGC_50_ are below 1 mg/L, a substance is classified as “very toxic to aquatic organisms” (danger symbol N, risk phrase R50). If the values obtained for toxicity are between 1 and 10 mg/L, a substance is classified as “toxic to aquatic organisms” (danger symbol N, risk phrase R51). A substance is classified as “harmful to aquatic organisms” if the end points obtained are between 10 and 100 mg/L (risk phrase R52). Classification is also based on the assessment of ready biodegradability or bioaccumulation potential (i.e., bioconcentration factor, BCF). If BCF ≥100, a compound is classified as “may cause long-term adverse effects in the aquatic environment” (risk phrase R53) [64,65]. The acute toxicity estimate (ATE) categories, identified by the CLP regulation, depend on the Oral rat LD_50_ [66]. The four ATE thresholds are; a) category 1, ATE ≤ 5 mg/Kg, a substance is classified as “Fatal if swallowed”, b) category 2, 5 < ATE ≤ 50 mg/Kg, a substance is classified as “Fatal if swallowed”, c) category 3, 50 < ATE ≤ 300 mg/Kg, a substance is classified as “Toxic if swallowed”, d) category 4, 300 < ATE ≤ 2000 mg/Kg, a compound is classified as “Harmful if swallowed” and e) category 5, ATE > 2000 mg/Kg, a substance is classified as “may be Harmful if swallowed”. Mutagenicity, carcinogenicity and reproductive toxicity are some of the most important endpoints to evaluate toxicity towards humans. Mutagenic toxicity can be experimentally assessed by various test systems; the most common is the Ames test, which makes use of a genetically engineered *Salmonella typhimurium* and *Esherichia coli* bacterial strains [67].

## 4. Conclusions

The bioprospection of marine-derived actinomycetes revealed napyradiomycins as potential antifouling agents. Given their potent antibacterial, antibiofilm, and antisettlement activities, they revealed the capacity to prevent growth or the primary adhesion of marine bacterial species to submersed abiotic surfaces as well as other subsequent fouling steps. Napyradiomycin (**1**) exhibited the higher antibacterial activity, (**4**) the higher microfouling inhibitory activity, and (**10**) the most potent antimacrofouling activity. However, napyradiomycins (**8** and **11**) would be our first choice for the development of antifouling paints, as they were active against all the assayed marine organisms. This broad spectrum activity is a clear advantage towards the commercialized products, including ivermictin and CuSO_4_, which are more limited, as they are directed for macrofouling inhibition only. Overall, in silico toxicity predictions of napyradiomycins suggest toxicity similar to marketed drugs and antifouling biocides, low bioaccumulation factor, and no mutagenicity. Taken together with the absence of toxic effects against the assayed species, napyradiomycins could be considered for further investigation as active ingredients for the marine antifouling paints and coatings development pipeline. In particular, the 3-chloro-napyradiomycin scaffold is indicated to be a key functional moiety for micro and macrofouling activities, especially having a bromine substitute at position C-16, such as (**8** and **11**). The correlation between napyradiomycin´s biosynthetic features and antifouling activities (micro and macro) opens prospects for their engineered biosynthetic enhanced yield production and future commercialization. 

## Figures and Tables

**Table 1:** Percentage of growth inhibition of different marine bacteria with the addition of napyradiomycins (**1**), (**4**), and (**8** and **11**). Shown are the average values of the percentage of growth inhibition of three replicates with the standard error of the mean (SEM). N.I.—not inhibited.

Napyradiomycin	(1)			(4)	(8 and 11)	
Concentration (µg/mL)	*M. luteus*	*C. marina*	*P. batsensis*	*M. luteus*	*M. luteus*	*C. marina*
15.60	96.5 ± 0.2	15.3 ± 0.7	62.7 ± 0.6	60.1 ± 8.5	93.9 ± 1.3	17.4 ± 0.5
7.81	96.0 ± 0.3	18.2 ± 0.7	47.4 ± 0.3	53.3 ± 5.7	96.8 ± 1.4	16.0 ± 1.2
3.91	93.6 ± 1.1	5.5 ± 0.8	N.I.	61.2 ± 3.7	95.1 ± 1.5	8.3 ± 5.1
1.95	69.8 ± 0.5	N.I.	N.I.	51.7 ± 4.5	82.7 ± 2.9	N.I.
0.98	42.9 ± 0.4	N.I.	N.I.	38.7 ± 1.9	70.5 ± 1.9	N.I.

**Table 2:** Results from the biofilm inhibition assay performed using napyradiomycins (**1**–**12**) at a concentration of 31.25 µg/mL. Shown are the average values of the percentage of biofilm inhibition of three replicates with the standard error of the mean (SEM). N.I.—not inhibited, N.T.—not tested.

	% Biofilm Inhibition ± SEM				
Napyradiomycin	*M. hydrocarbonoclasticus*	*C. marina*	*M. luteus*	*P. batsensis*	*P. inhibens*
(**1**)	N.I.	N.I.	88.6 ± 2.9	87.2 ± 0.1	N.I.
(**2**)	60.2 ± 5.7	N.I.	N.I.	N.I.	N.I.
(**4**)	56.9 ± 5.7	N.I.	97.0 ± 1.2	13.4 ± 0.8	N.I.
(**5** and **6**)	43.4 ± 8.2	N.I.	87.3 ± 0.3	N.I.	N.I.
(**8** and **11**)	59.3 ± 3.8	N.I.	100 ± 0.3	26.2 ± 0.0	N.I.
(**10**)	25.1 ± 10.2	N.I.	N.T.	N.T.	N.T.
(**12**)	33.7 ± 5.1	N.I.	N.T.	N.T.	N.T.
(**3** and **7**)	28.0 ± 4.8	23.4 ± 4.9	N.T.	N.T.	N.T.
(**9** and **12**)	48.2 ± 1.9	N.I.	N.T.	N.T.	N.T.

**Table 3:** Percentage of inhibition of biofilm formation for different marine bacteria in the presence of different concentrations of napyradiomycins (**1)**, (**2)**, (**4**), (**5** and **6**)**,** and (**8** and **11**). Shown are the average values of the percentage of biofilm inhibition of three replicates with the standard error of the mean (SEM). N.I.—not inhibited.

Napyradiomycin	(1)		(2)	(4)		(5 and 6)		(8 and 11)		
Concentration (µg/mL)	*M. luteus*	*P. batsensis*	*M. hydro.*	*M. hydro.*	*M. luteus*	*M. hydro.*	*M. luteus*	*M. hydro.*	*M. luteus*	*P. batsensis*
**15.6**	95.4 ± 0.9	86.9 ± 1.0	56.6 ± 0.9	82.3 ± 1.5	88.8 ± 4.2	38.8 ± 8.4	80.1 ± 6.5	62.8 ± 4.0	100 ± 0.3	37.8 ± 0.7
**7.81**	96.7 ± 0.5	28.4 ± 2.8	51. 7± 2.0	67.0 ± 5.5	86.7 ± 3.0	53.2 ± 8.2	65.9 ± 12.3	64.9 ± 3.0	100 ± 0.8	23.1 ± 0.2
**3.91**	95.8 ± 2.9	38.1 ± 0.6	67. 7± 1.2	84.5 ± 2.0	100 ± 0.4	43.8 ± 9.4	82.5 ± 5.1	72.5 ± 2.3	100 ± 0.6	7.1 ± 4.2
**1.95**	90.3 ± 3.8	17.7 ± 0.8	56.5 ± 5.7	78.1 ± 3.0	91.7 ± 0.9	44.1 ± 9.6	32.2 ± 13.9	52.7 ± 10.2	100 ± 0.3	7.9 ± 1.0
**0.98**	59.0 ±7.4	4.3 ± 4.3	24.6 ± 9.8	56.1 ± 5.6	93.8 ± 4.1	48.2 ± 6.4	33.3 ± 7.4	66.3 ± 6.9	100 ± 0.9	N.I.

**Table 4:** Response of *M. galloprovincialis* plantigrade larvae settlement following incubation with napyradiomycins (**1**–**12**), after a 15 h acute exposure assay. Pearson goodness-of-fit (Chi-Square-χ^2^) significance was considered at *p* < 0.05 and 95% lower and upper confidence limits (95% LCL; UCL) were presented. Therapeutic ratio (LC_50_/EC_50_) was used to evaluate the effectiveness of each compound vs. its toxicity. Negative control: dimethylsulphoxide (DMSO) = 100% settlement; Positive control: CuSO_4_ 0.16 μg/mL (5 μM) = 0% settlement.

Napyradiomycin	EC_50_ [Conf. limits] (µg/mL)	Chi-Square Test	LC_50_ (µg/mL)	LC_50_/EC_50_
(**1**)	0.655 [0.300; 0.906]	χ^2^ = 217.986; *df* = 18; *p* < 0.001	>12	18.32
(**2**)	1.999 [1.581; 2.547]	χ^2^ = 414.500; *df* = 18; *p* < 0.001	>12	6.00
(**3** and **7**)	1.092 [0.225; 2.933]	χ^2^ = 555.409; *df* = 18; *p* < 0.001	>12	10.99
(**4**)	6.339 [5.602; 7.181]	χ^2^ = 144.409; *df* = 18; *p* < 0.001	>12	1.89
(**5** and **6**)	4.331 [2.911; 7.091]	χ^2^ = 617.072; *df* = 18; *p* < 0.001	>12	2.77
(**8** and **11**)	0.727 [0.065; 1.406]	χ^2^ = 458.713; *df* = 18; *p* < 0.001	>12	16.51
(**9** and **12**)	0.451 [0.192; 0.760]	χ^2^ = 770.695; *df* = 22; *p* < 0.001	>12	26.58
(**10**)	0.102 [0.072; 0.140]	χ^2^ = 844.065; *df* = 42; *p* < 0.001	>12	117.28
(**12**)	0.947 [0.586; 1.473]	χ^2^ = 729.107; *df* = 22; *p* < 0.001	>12	12.67

**Table 5:** Toxicity end point predictions for napyradiomycins **1**–**12**.

Toxicity End Points for Consensus Models							
#	Fathead Minnow ^1^	*Daphnia magna ^2^*	*Tetrahymena pyriformis ^3^*	Oral Rat ^4^	Bioconcentration Factor	Developmental Toxicity ^5^	Ames Mutagenicity ^6^
**1**	0.27	0.76	3.01	495.72	22.17	0.88; DT	0.23; MN
**2**	0.22	0.45	1.22	291.93	10.25	0.72; DT	0.17; MN
**3**	0.05	0.17	310	505.42	42.17	0.71; DT	0.31; MN
**4**	0.13	1.64	1.22	491.94	5.31	0.51; DT	0.36; MN
**5**	0.05	3.23	3.09	478.12	26.41	0.73; DT	0.15; MN
**6**	0.09	2.28	2.87	1246.67	10.36	0.92; DT	0.18; MN
**7**	0.06	1.23	2.87	895.08	26.62	0.96; DT	0.17; MN
**8**	0.04	0.41	1.65	1055.66	18.59	0.95; DT	0.27; MN
**9**	0.04	0.98	1.56	1516.88	22.55	0.91; DT	0.25; MN
**10**	0.63	0.91	1.62	390.57	36.37	0.74; DT	0.29; MN
**11**	0.02	0.38	5.61	1414.33	31.86	0.93; DT	0.19; MN
**12**	0.05	0.45	5.29	687.01	68.24	0.90; DT	0.19; MN

^1^ 96 hour LC_50_ (mg/L). ^2^ 48 hour LC_50_ (mg/L). ^3^ 48 hour IGC_50_ (mg/L), the Nearest Neighbor model, the other models are unable to predict this end point. ^4^ LD_50_ (mg/kg). ^5^ DT: developmental toxicant. ^6^ MN: mutagenicity negative.

## Supplementary Materials

The following are available online at https://www.mdpi.com/1660-3397/18/1/63/s1, napyradiomycins (**1**–**12**) structural characterization description; Table S1. Toxicity end point predictions for seven Prestwick approved drugs; Table S2. Toxicity end point predictions for two antifouling approved drugs; Table S3. Aquatic toxicity, environmental fate data and classification of copper and arsenic, experimental data.
