# Supplementary material for: Antifouling Napyradiomycins from Marine-Derived Actinomycetes Streptomyces aculeolatus
Source: Mar Drugs. 2020 Jan 18;18(1):63. doi: 10.3390/md18010063 (PMC7024211; doi:10.3390/md18010063)
Supplement: Supplementary file 1 [file marinedrugs-18-00063-s001.pdf]

marine drugs

MDPI

Supplementary Materials

Antifouling napyradiomycins from marine-derived actinomycetes *Streptomyces aculeolatus*

Florbela Pereira[1], Joana R. Almeida[2], Marisa Paulino[3], Inês R. Grilo[4], Helena Macedo[3,4], Isabel Cunha[2], Rita G. Sobral[4], Vitor Vasconcelos[2,5] and Susana P. Gaudêncio[1,3*]

[1] LAQV, Chemistry Department, Faculty for Sciences and Technology, NOVA University of Lisbon, 2829-516 Caparica, Portugal.

[2] CIIMAR/CIMAR - Interdisciplinary Centre of Marine and Environmental Research, University of Porto, Terminal de Cruzeiros do Porto de Leixões, Avenida General Norton de Matos, 4450-208 Matosinhos, Portugal.

[3] UCIBIO, Chemistry Department, Blue Biotechnology and Biomedicine Lab, Faculty for Sciences and Technology, NOVA University of Lisbon, 2829-516 Caparica, Portugal.

[4] Life Sciences Department, UCIBIO, Faculty for Sciences and Technology, NOVA University of Lisbon, 2829-516 Caparica, Portugal.

[5] Biology Department, Faculty of Sciences, Porto University, Rua do Campo Alegre, 4069-007, Porto, Portugal.

* Correspondence: s.gaudencio@fct.unl.pt (S.P.G.)

1. Structural characterization of napyradiomycin derivatives

The molecular formulas of **1** and **2** were established by HR-MS as $C_{25}H_{30}{}^{35}Cl_2O_5$ (*m/z* 479.1400 [M - H]$^-$, calcd 479.1398) and $C_{25}H_{30}{}^{35}Cl_2O_6$ (*m/z* 495.1349 [M - H]$^-$, calcd 495.1347) respectively. The ^{1}H and ^{13}C NMR data of **1** and **2** are similar to those of napyradiomycin A1 (**1**) produced by *Streptomyces* spp. [1,2] and 18-hydroxynapyradiomycin A1 (**2**) produced by *S. antimycoticus* [3] respectively. NMR data for compound **1** and **2** are very similar, the ^{1}H NMR spectrum of **1** and **2** clearly presented one exchangeable OH signal (δ_H 11.98 ppm in **1** and **2**), two single aromatic protons (δ_H 7.22, 6.74 ppm in **1** and 7.13, 6.71 ppm in **2**), two olefinic proton signals at δ_H 4.89, 4.71 ppm in **1** and 5.28, 4.90 ppm in **2**, one methine proton adjacent to chlorine at δ_H 4.42 ppm in **1** and 4.47 ppm in **2**, three methylene proton signals (δ_H 2.46, 1.64 ppm in **1** and 2.52, 2.41, 1.89 ppm in **2**), and four methyl groups at δ_H 1.50, 1.31, 1.18 ppm in **1** and 1.64, 1.50, 1.23 ppm in **2**. The ^{13}C NMR spectrum of **1** and **2** showed two carbonyl signals at δ_C 196.27, 193.78 ppm in **1** and 194.68, 193.72 ppm in **2**, two phenolic hydroxyl groups (δ_C 167.68, 164.73 ppm in **1** and 165.08, 165.28 ppm in **2**), four aromatic carbon signals resonating between δ_C 135.16 and 107.77 ppm in **1** and between δ_C 134.80 and 108.20 ppm in **2**, four sp^2 carbon signals resonating between δ_C 142.85 and 114.89 ppm in **1** and between δ_C 139.70 and 116.87 ppm in **2**, two quaternary carbon adjacent to oxygen atom (δ_C 83.51, 78.93 ppm in **1** and 84.46, 78.86 ppm in **2**), and one methylene carbon adjacent to chlorine atom (δ_C 58.74 in **1** and **2**), as well as other seven aliphatic methylene or methyl carbon signals with chemical shifts were below δ_C 50.0 ppm in **1** and **2**. The main differences between **1** and **2** in the ^{1}H NMR is that the signal of the 17-Me methyl singlet (δ_H 1.50 ppm) in **1** was replaced by methylene protons (δ_H 4.19 and 4.09 ppm, d, J = 12. 7 Hz) in **2**. The ^{13}C NMR spectra of **1** and **2** showed the presence of a methyl carbon (δ_C 17.54 ppm for 17-CH_3) and an oxymethylene carbon (δ_C 69.10 ppm for 17-CH_2OH), respectively.

Mass spectrometry data obtained by HR-MS confirmed the presence of two compounds, the molecular formulas of **3** and **7** were established as $C_{26}H_{32}{}^{35}Cl_2O_5$ (*m/z* 493.1548 [M - H]$^-$, calcd 493.1549) and $C_{26}H_{31}{}^{35}ClO_5$ (*m/z* 457.18 [M - H]$^-$, calcd 457.1782) respectively. The comparison of ^{1}H NMR data of **3** and **7** revealed that **7** only differed from **3** by having an olefinic bond at C-4 and C-4a, which was evident from the presence of an alkene proton signal at δ_H 6.92 ppm (d, J = 1.6 Hz, H-4 in **7**). Thus, **3** and **7** were established as SF2415B3 [4] (**3**) and 4-dehydro-4a-dechloro-SF2415B3 (**7**) respectively. Compounds **3** and **7** co-eluted, taking into account the integration of the exchangeable 6-OH signals (δ_H 12.90 ppm in **3** and δ_H 12.14 ppm in **7**) it was possible to establish the ratio of the two compounds in the mixture, which is approximately 67:33 of **3** and **7**. ^{1}H and ^{13}C NMR data of **3** are very similar to that of **1**, discussed above, the ^{1}H and ^{13}C NMR spectra of **3** showed that one aromatic proton of **1** (δ_H 6.74 ppm) was substituted by one methyl group in **3** (δ_H 2.23 ppm and δ_C 8.1 ppm).

The molecular formula of **4** was established as $C_{25}H_{30}{}^{35}Cl_2O_6$ by HR-MS (*m/z* 495.1346 [M - H]$^-$, calcd 495.1347). The ^{1}H and ^{13}C NMR data of **4** were very similar and indistinguishable from the known compound napyradiomycin A2 (**4**) [5]. The hydroxyl substituent at C-16 was confirmed by the ^{13}C and ^{1}H NMR chemical shifts of 75.39 ppm and 4.03 ppm (t, J = 6.4 Hz) respectively.

Mass spectrometry data obtained by HR-ESI-MS confirmed the presence of two compounds, the molecular formulas of **5** and **6** were established as $C_{25}H_{28}{}^{35}Cl_2O_6$ (*m/z* 493.1197 [M - H]$^-$, calcd 493.1190) and $C_{25}H_{27}{}^{35}ClO_6$ (*m/z* 457.1429 [M - H]$^-$, calcd 457.1423) respectively. The comparison of ^{1}H NMR data of **5** and **6** revealed that **6** only differed from **5** by having an olefinic bond at C-4 and C-4a, which was evident from the presence of an alkene proton signal at δ_H 6.40 ppm (br s, H-4 in **5**), and the absence of the methylene proton signal at δ_H 2.55 ppm (m, two H-4 in **5**). Thus, **5** and **6** were established as 16-oxonapyradiomycin A2 (**5**) [3] and 4-dehydro-4a-dechloro-16-oxonapyradiomycin A2 (**6**) respectively. Compounds **5** and **6** co-eluted, taking into account the

integration of the exchangeable 6-OH signals (δ_H 11.88 ppm in **5** and δ_H 11.83 ppm in **6**) it was possible to establish the ratio of the two compounds in the mixture, which is approximately 60:40 of **5** and **6**.

Mass spectrometry data obtained by HR-MS confirmed the presence of two compounds, the molecular formulas of **8** and **11** were established as $C_{25}H_{29}{}^{79}Br^{35}Cl_2O_5$ (*m/z* 557.0503 [M - H]$^-$, calcd 557.0503) and $C_{25}H_{28}{}^{79}Br^{35}ClO_5$ (*m/z* 521.0738 [M - H]$^-$, calcd 521.0736) respectively. The comparison of ^{1}H NMR data of **8** and **11** showed that **11** only differed from **8** by having an olefinic bond at C-4 and C-4a, which was evident from the presence of an alkene proton signal at δ_H 6.87 ppm (d, J = 1.7 Hz, H-4 in **11**), and the lack of the methylene proton signal at δ_H 2.52 ppm (dd, J = 13.9 and 3.9 Hz, two H-4 in **8**). In the same way, the ^{13}C NMR spectrum of **11** showed two olefinic carbon atoms for C-4 (δ_C 137.02 ppm, CH) and C-4a (δ_C 136.9 ppm, qC). Whereas in the ^{13}C NMR spectrum of **8**, there was a methylene carbon at C-4 (δ_C 42.81 ppm, CH_2) and a quaternary carbon at C-4a (δ_C 78.91 ppm, qC). Therefore, **8** and **11** were established as napyradiomycin B3(**8**) [2] and 4-dehydro-4a-dechloro-napyradiomycin B3 (**11**) [6] respectively. Similar to compounds **5** and **6**, the napyradiomycins **8** and **11** co-eluted, taking into account the integration of the exchangeable 6-OH signals (δ_H 12.04 ppm in **8** and δ_H 12.58 ppm in **11**) it was possible to establish the ratio of the two compounds in the mixture, which was approximately 56:44 of **8** and **11**.

Mass spectrometry data obtained by HR-MS confirmed the presence of two compounds, the molecular formulas of **9** and **12** were established as $C_{26}H_{31}{}^{35}Cl_3O_5$ (*m/z* 527.1170 [M - H]$^-$, calcd 527.1164) and $C_{26}H_{30}{}^{35}Cl_2O_5$ (*m/z* 491.23 [M - H]-, calcd 491.1398) respectively. The comparison of ^{1}H NMR data of **9** and **12** revealed that **12** only differed from **9** by having an olefinic bond at C-4 and C-4a, which was evident from the presence of an alkene proton signal at δ_H 6.86 ppm (d, J = 1.6 Hz, H-4 in **12**), and the absence of the methylene proton signal at δ_H 2.54 ppm (m, two H-4 in **9**). Comparison of ^{1}H and ^{13}C NMR data of **8** and **9**, as well as **11** and **12** showed that in addition to the substitution of the aromatic proton H-7 of compounds **8** and **11** by the methyl group 7-CH_3 in the compounds **9** and **12**, the pairs of compounds **8**, **9** and **11**, **12** differ only from each another by having at the 16-position a bromine atom (**8** and **11**) or a chlorine atom (**9** and **12**) respectively. Taking into account the ^{13}C NMR data, it showed a shift of the C-16 signal from 66.85 ppm and 66.43 ppm in compounds **8** and **11** respectively to 70.70 ppm and 70.87 ppm in compounds **9** and **12** respectively. Thus, **9** and **12** were established as A80915A (**9**) [7] and 4-dehydro-4a-dechloro-A80915A(**12**) [8] respectively. Compounds **9** and **12** also co-eluted, taking into account the integration of the exchangeable 6-OH signals (δ_H 12.90 ppm in **9** and δ_H 12.36 ppm in **12**) it was possible to establish the ratio of the two compounds in the mixture, which is approximately 65:35 of **9** and **12**.

The molecular formula of **10** was established as $C_{26}H_{33}{}^{35}Cl_3O_6$ by HR-MS (*m/z* 545.1270 [M - H]$^-$, calcd 545.1270). The ^{1}H and ^{13}C NMR data of **10** were very similar and indistinguishable from the known compound A80915C (**10**) [7,9]. The hydroxyl and methyl substituents at C-13 were confirmed by the ^{13}C and ^{1}H NMR chemical shifts of 72.24 ppm (qC, C-13), 24.38 ppm (CH_3, 13-Me), 6.52 ppm (s, 13-OH) and 1.33 ppm (s, CH_3, 13-Me) respectively.

2. Data for *structural characterization of napyradiomycin derivatives*

Napyradiomycin A1 (1):

Orange oil (12.86 mg); **UV** λ_{max} **(nm):** 200.5, 251.1, 360.4; **IR NaCl** ν_{max} **(cm^{-1}):** 3341.13, 1614.99, 1450.10, 2984.33, 2929.27, 759.56; **^{1}H NMR (400 MHz, $CDCl_3$):** δ 11.98 (s, 1H, 6-OH), 7.22 (d, 1H, *J*= 1.8 Hz, H-9), 6.74 (d, 1H, *J*= 1.8 Hz, H-7), 4.89 (br s, 1H, H-16), 4.71 (t, 1H, *J*= 8.1 Hz, H-12), 4.42 (dd, 1H, *J*= 11.4, 4.5 Hz, H-3), 2.70 (d, 2H, *J*= 8.2 Hz, H-11), 2.46 (m, 2H, H-4), 1.62 (s, 3H, 17-Me), 1.64 (m,

4H, H-14, H-15), 1.50 (s, 6H, 2-Me, 17-Me), 1.31 (s, 3H, 13-Me), 1.18 (s, 3H, 2-Me); **^{13}C NMR (100 MHz, $CDCl_3$):** δ 196.27 (Cq, C-10), 193.78 (Cq, C-5), 167.68 (Cq, C-6), 164.73 (Cq, C-8), 142.85 (Cq, C-13), 135.16 (Cq, C-9a), 131.76 (Cq, C-17), 123.67 (CH, C-16), 114.89, (CH, C-12), 110.20 (Cq, C-5a), 109.57 (CH, C-7), 107.77 (CH, C-9), 83.51 (Cq, C-10a), 78.93 (Cq, C-2), 78.78 (Cq, C-4a), 58.74 (CH, C-3), 42.68 (CH_2, C-4), 41.29 (CH_2, C-11), 39.72 (CH_2, C-14), 28.74 (CH_3, 2-Me), 25.91 (CH_2, C-15), 25.63 (CH_3, 17-Me), 22.24 (CH_3, 2-Me), 17.54 (CH_3, 17-Me), 16.44 (CH_3, 13-Me); **HR-MS *m/z*** 479.1400 (calcd for $C_{25}H_{29}{}^{35}Cl_2O_5$, $[M-H]^-$ 479.1398); **R_T:** 71.9 min.

18-hydroxynapyradiomycin A1 (2):
Orange oil (5.04 mg); **UV λ_{max} (nm):** 197.7, 252.4, 361.3; **IR NaCl ν_{max} (cm^{-1}):** 3100, 1615.34, 1259.18, 757.79; **^{1}H NMR (400 MHz, $CDCl_3$):** δ 11.98 (s, 1H, 6-OH), 7.13 (br s, 1H, H-9), 6.71 (br s, 1H, H-7), 5.28 (m, 1H, H-16), 4.90 (t, 1H, *J*= 7.7 Hz, H-12), 4.47 (m, 1H, H-3), 4.19 (d, 1H, *J*= 12.7 Hz, 17-CH_2OH), 4.09 (d, 1H, *J*= 12.7 Hz, 17-CH_2OH), 2.73 (m, 1H, H-11), 2.52 (m, 2H, H-4, H11), 2.41 (m, 1H, H-4), 1.89 (m, 4H, H-14, H-15), 1.64 (s, 3H, 17-Me), 1.50 (s, 3H, 2-Me), 1.23 (s, 6H, 2-Me,13-Me); **^{13}C NMR (100 MHz, $CDCl_3$):** δ 194.68 (Cq, C-10), 193.72 (Cq, C-5), 165.28 (Cq, C-8), 165.08 (Cq, C-6), 139.70 (Cq, C-13), 134.80 (Cq, C-9a), 133.69 (Cq, C-17), 126.69 (CH, C-16), 116.87 (CH, C-12), 109.47 (CH, C-7), 108.9 (Cq, C-5a), 108.20 (CH, C-9), 84.46 (Cq, C-10a), 79.07 (Cq, C-4a), 78.86 (Cq, C-2), 69.10 (CH_2, 17-CH_2OH), 58.74 (CH, C-3), 42.41 (CH_2, C-4), 40.48 (CH_2, C-11), 38.67 (CH_2, C-14), 29.08 (CH_3, 2-Me), 24.81 (CH_2, C-15), 22.22 (CH_3, 2-Me), 15.73 (CH_3, 13-Me), 13.78 (CH_3, 17-Me); **HR-MS *m/z*** 495.1349 (calcd for $C_{25}H_{29}{}^{35}Cl_2O_6$, $[M-H]^-$ 495.1347); **R_T:** 36.5 min.

Napyradiomycin SF2415B3 (3):
Orange oil (6.80 mg); **UV λ_{max} (nm):** 196.2, 263.9, 323.3; **IR NaCl ν_{max} (cm^{-1}):** 3293.88, 1698.70, 1608.31, 1433.37, 2981.60, 2927.53, 757.16; **^{1}H NMR (400 MHz, $CDCl_3$):** δ 12.90 (s, 1H, 6-OH), 7.27 (s, 1H, H-9), 5.03 (br s, 1H, H-16), 4.89 (t, 1H, *J*= 8.1 Hz, H-12), 4.42 (dd, 1H, *J*= 11.3, 4.4 Hz, H-3), 2,70 (br d, 2H *J*= 8.1 Hz, H-11), 2.46 (m, 2H, H-4), 2.23 (s, 3H, 7-Me), 1.70 (s, 3H, 17-Me), 1.64 (m, 4H, H-14, H-15), 1.59 (s, 3H, 17-Me), 1.55 (s, 3H, 2-Me), 1.34 (s, 3H, 13-Me), 1.09 (s, 3H, 2-Me); **HR-MS *m/z*** 493.1548 (calcd for $C_{26}H_{32}{}^{35}Cl_2O_5$, $[M-H]^-$, 493.1549); **R_T:** 37.6 min.

Napyradiomycin A2 (4):
Orange oil (11.36 mg); $[\alpha]_D$ –19 (*c* 0.42, $CHCl_3$); **UV λ_{max} (nm):** 197.9, 251.8, 361.1; **IR NaCl ν_{max} (cm^{-1}):** 3300, 1615.34, 757.79, 1386.13, 1371.64; **^{1}H NMR (400 MHz, $CDCl_3$):** δ 11.94 (s, 1H, 6-OH), 7.32 (d, 1H, *J*= 1.6 Hz, H-9), 6.75 (br s, 1H, H-7), 4.96 (s, 1H, H-18a), 4.87 (s, 1H, H-18b), 4.76 (t, 1H, *J*= 7,8 Hz, H-12), 4.45 (dd, 1H, *J*= 11.8 Hz, *J*= 3.8 Hz, H-3), 4.03 (t, 1H, *J*= 6.4 Hz, H-16), 2.81 (m, 1H, H-11), 2.48 (m, 3H, H-4, H-11), 1.82 (t, 2H, *J*= 7.4 Hz, H-14), 1.71 (s, 3H, 17-Me), 1.51 (s, 3H, 2-Me), 1.35 (s, 3H, 13-Me), 1.34 (m, 2H, H-15), 1.19 (s, 3H, 2-Me); **^{13}C NMR (100 MHz, $CDCl_3$):** δ 196.13 (qC, C-10), 193.70 (qC, C-5), 165.24 (qC, C-6, C-8), 146.46 (qC, C-17), 141.54 (qC, C-13), 134.76 (qC, C-9a), 116.52 (CH, C-12), 111.97 (CH_2, C-18), 110.16 (CH, C-7), 109.39 (qC, C-5a), 108.52 (CH, C-9), 84.30 (qC, C-10a), 79.26 (qC, C-2), 79.10 (qC, C-4a), 75.39 (CH, C-16), 58.77 (CH, C-3), 42.69 (CH_2, C-4), 40.22 (CH_2, C-11), 35.86 (CH_2, C-14), 31.85 (CH_2, C-15), 29.05 (CH_3, 2-Me), 22.38 (CH_3, 2-Me), 17.88 (CH_3, 17-Me), 16.11 (CH_3, 13-Me); **HR-MS *m/z*** 495.1346 (calcd for $C_{25}H_{29}{}^{35}Cl_2O_6$, $[M-H]^-$, 495.1347; **R_T:** 40.3 min.

16-oxonapyradiomycin A2 (5):
Orange oil (1.67 mg); **UV λ_{max} (nm):** 198.0, 234.48, 361.5; **IR NaCl ν_{max} (cm^{-1}):** 3400, 2980.84, 2928.02, 1615.54, 1257.31, 758.11; **^{1}H NMR (400 MHz, $CDCl_3$):** δ 11.88 (s, 1H, 6-OH), 9.37 (s, 1H, 8-OH), 7.10 (s, 1H, H-9), 6.73 (s, 1H, H-7), 6.41 (s, 1H, H-18), 5.79 (s, 1H, H-18), 4.84 (t, 1H, *J*= 7.2 Hz, H-12), 4,43 (dd, 1H, *J*= 11.7, 4.5 Hz, H-3), 2,68 (br d, 2H *J*= 8.2 Hz, H-11), 2.55 (m, 2H, H-4), 2.40 (m, 2H, H-15),

1.97 (m, 2H, H-14), 1.67 (s, 3H, 17-Me), 1.50 (s, 3H, 2-Me), 1.37 (s, 3H, 13-Me), 1.19 (s, 3H, 2-Me); **HR-MS *m*/*z*** 493.1197 (calcd for $C_{25}H_{27}{}^{35}Cl_2O_6$, $[M-H]^-$, 493.1190), **R_T:** 54.4 min.

4-dehydro-4a-dechloro-16-oxonapyradiomycin A2 (6):
Orange oil (1.67 mg); **UV λ_{max} (nm):** 198.0, 234.48, 361.5; **IV NaCl ν_{max} (cm⁻¹):** 3400, 2980.84, 2928.02, 1615.54, 1257.31, 758.11; **¹H NMR (400 MHz, $CDCl_3$):** δ 11.83 (s, 1H, 6-OH), 7.20 (s, 1H, H-9), 7.10 (s, 1H), 6.70 (s, 1H, H-7), 6.40 (br s, 1H, H-4), 6.38 (s, 1H, H-18), 5.90 (s, 1H, H-18), 4.73 (t, 1H, *J*= 7.5 Hz, H-12), 4.40 (br s, 1H, H-3), 2.75 (dd, 2H, *J*= 14.2, 7.5 Hz, H-12), 2.48 (m, 2H, H-15), 2,15 (m, 2H, H-14), 1.84 (s, 3H, 17-Me), 1.50 (s, 3H, 2-Me), 1.34 (s, 3H, 13-Me), 1.18 (s, 3H, 2-Me); **HR-MS *m*/*z*** 457.1429 (calcd for $C_{25}H_{26}{}^{35}ClO_6$, $[M-H]^-$, 457.1423); **R_T:** 54.4 min.

4-dehydro-4a-dechloro-16-oxo napyradiomycin SF2415B3 (7):
Orange oil (6.80 mg); **UV λ_{max} (nm):** 196.2, 263.9, 323.3; **IR NaCl ν_{max} (cm⁻¹):** 3293.88, 1698.70, 1608.31, 1433.37, 2981.60, 2927.53, 757.16; **¹H NMR (400 MHz, $CDCl_3$):** δ 12.14 (s, 1H, 6-OH), 7.23 (s, 1H, H-9), 6.92 (d, 1H, *J*= 1.6 Hz, H-4), 5.01 (br s, 1H, H-16), 4.71 (t, 1H, *J*= 8.0 Hz, H-12), 4.39 (d, 1H, *J*= 1.6 Hz, H-3), 2.22 (s, 3H, 7-Me), 1.70 (s, 3H, 17-Me), 1.64 (m, 4H, H-14, H-15), 1.59 (s, 3H, 17-Me), 1.55 (s, 3H, 2-Me), 1.33 (s, 3H, 13-Me), 1.18 (s, 3H, 2-Me); **HR-MS *m*/*z*** 457.18 (calcd for $C_{26}H_{31}{}^{35}ClO_5$, $[M-H]^-$, 457.1782); **R_T:** 37.6 min.

Napyradiomycin B3 (8):
Orange oil (5.21 mg); **UV λ_{max} (nm):** 196.1, 252.2, 357.6; **IV NaCl ν_{max} (cm⁻¹):** 3321.45, 2933.19, 2980.36, 1703.09, 1619.64, 1260.41, 756.03; **¹H NMR (400 MHz, $CDCl_3$):** δ 12.04 (s, 1H, 6-OH), 7.14 (d, 1H, *J*= 1.7 Hz, H-9), 6.74 (br s, 1H, H-7), 4.78 (br s, 1H, 13-CH_2), 4.76 (br s, 1H, 13-CH_2), 4.45 (dd, 1H, *J*= 11.1, 3.9 Hz, H-3), 4.06 (dd, 1H, *J*= 10.8, 4.1 Hz, H-16), 2.66 (dd,1H, *J*= 15.2, 8.6 Hz, H-12), 2.52 (dd, 2H, *J*= 13.9, 3,9 Hz, H-4), 2,20 (m, 2H, H-14, H-15), 2.04 (m, 1H, H-11), 1.93 (m, 2H, H-14, H-15), 1.61 (br d, 2H, *J*= 15 Hz, H-11), 1.37 (s, 3H, 2-Me), 1.19 (s, 3H, 2-Me), 0.72 (s, 3H, 17-Me), 0.66 (s, 3H, 17-Me); **¹³C NMR (100 MHz, $CDCl_3$):** δ 195.05 (qC, C-10), 193.57 (qC, C-5), 165.58 (qC, C-8), 163.83 (qC, C-6), 145.70 (qC, C-13), 135.16 (qC, C-9a), 110.35 (CH_2, 13-CH_2), 109.58 (qC, C-5a), 109.17 (CH, C-7), 108.64 (CH, C-9), 84.36 (qC, C-10a), 81.01 (qC, C-2), 78.91 (qC, C-4a), 66.85 (CH, C-16), 58.87 (CH, C-3), 45.84 (CH, C-12), 42.81 (CH_2, C-4), 41.94 (qC, C-17), 37.44 (CH_2, C-14), 36.03 (CH_2, C-15), 35.47 (CH_2, C-11), 29.09 (CH_3, 2-Me), 27.92 (CH_3, 17-Me), 22.55 (CH_3, 2-Me), 16.45 (CH_3, 17-Me); **HR-MS *m*/*z*** 557.0503 (calcd for $C_{25}H_{28}{}^{79}Br^{35}Cl_2O_5$, $[M-H]^-$, 557.0503); **R_T:** 81.5 min.

Napyradiomycin A80915A (9):
Orange oil (7.40 mg); **UV λ_{max} (nm):** 193.2, 258.6, 314.6; **IR NaCl ν_{max} (cm⁻¹):** 3329.40, 1696.48, 1605.55, 1442.64, 2977.05, 2931.59, 760.67; **¹H NMR (400 MHz, $CDCl_3$):** δ 12.90 (s, 1H, 6-OH), 7.20 (s, 1H, H-9), 4.82 (br s, 1H, 13-CH_2), 4.44 (dd, 1H, *J*= 11.2, 4.0 Hz, H-3), 4.31 (br s, 1H, 13-CH_2), 3.83 (dd, 1H, *J*= 11.4, 4.2 Hz, H-16), 2.66 (dd,1H, *J*= 15.5, 8.5 Hz, H-12), 2.52 (dd, 2H, *J*= 14.0, 3,9 Hz, H-4), 2,33 (m, 2H, H-14, H-15), 2.24 (br s, 3H, 7-Me), 1.90 (m, 1H, H-11), 1.75 (m, 2H, H-14, H-15), 1.61 (br d, 2H, *J*= 15 Hz, H-11), 1.33 (s, 3H, 2-Me), 1.19 (s, 3H, 2-Me), 1.05 (s, 3H, 17-Me), 0.63 (s, 3H, 17-Me); **HR-MS *m*/*z*** 527.1170 (calcd for $C_{26}H_{31}{}^{35}Cl_3O_5$, $[M-H]^-$, 527.1164); **R_T:** 30.6 min.

Napyradiomycin A80915C (10):
Orange oil (13.2 mg); **UV λ_{max} (nm):** 194.9, 264.6, 326.6; **IV NaCl ν_{max} (cm⁻¹):** 3358.92, 1701.91, 1631.85, 1600.32, 1449.68, 2977.07, 2924.52, 756.05; **¹H NMR (400 MHz, $CDCl_3$):** δ 12.28 (s, 1H, 6-OH), 10.50 (s, 1H, 8-OH), 7.90 (s, 1H, H-9), 6.52 (s, 1H, 13-OH), 4.58 (dd, 1H, *J*= 11.9, 3.5 Hz, H-3), 3.44 (dd, 1H, *J*= 12.2, 3.5 Hz, H-16), 2.64 (dd, 1H, *J*= 14.2, 3.8 Hz, H-4), 2.51 (m, 1H, H-4), 2.51 (m, 1H, H-11), 2,23 (s, 3H, 7-Me), 1.94 (m, 2H, H-14, H-15), 1.78 (m, 1H, H-15), 1.59 (m, 1H, H-11), 1.44 (m,

2H, H-112, H14), 1.57 (s, 3H, 2-Me), 1.37 (s, 3H, 2-Me), 1.33 (s, 3H, 13-Me), 0.76 (s, 3H, 17-Me), 0.42 (s, 3H, 17-Me); **^{13}C NMR (100 MHz, $CDCl_3$):** δ 192.64 (qC, C-5), 190.96 (qC, C-10), 164.31 (qC, C-8), 163.33 (qC, C-6), 131.76 (qC, C-9a), 109.58 (qC, C-5a), 120.38 (qC, C-7), 108.65 (CH, C-9), 85.52 (qC, C-10a), 81.52 (qC, C-2), 80.36 (qC, C-4a), 72.24 (qC, C-13), 71.02 (CH, C-16), 57.90 (CH, C-3), 52.14 (CH, C-12), 42.08 (CH_2, C-4), 40.79 (CH_2, C-14), 40.54 (qC, C-17), 38.20 (CH_2, C-11), 29.83 (CH_2, C-15), 28.51 (CH_3, 2-Me), 28.51 (CH_3, 17-Me), 24.38 (CH_3, 13-Me), 22.97 (CH_3, 2-Me), 15.70 (CH_3, 17-Me), 8.43 (CH_3, 7-Me); **HR-MS *m*/*z*** 545.1270 (calcd for $C_{26}H_{33}{}^{35}Cl_3O_6$, $[M-H]^-$, 545.1270); **R_T:** 41.1 min.

4-dehydro-4a-dechloro-16-oxonapyradiomycin B3 (11):
Orange oil (5.21 mg); **UV λ_{max} (nm):** 196.1, 252.2, 357.6; **IV NaCl ν_{max} (cm^{-1}):** 243321.45, 2933.19, 2980.36, 1703.09, 1619.64, 1260.41, 756.03; **^{1}H NMR (400 MHz, $CDCl_3$):** δ 12.58 (s, 6-OH), 7.13 (br s, 1H, H-9), 6.87 (d, 1H, 1.7 Hz, H-4), 6,71 (br s, 1H, H-7), 4.82 (br s, 1H, 13-CH_2), 4.46 (d, 1H, *J*= 1.8 Hz, H-3), 4,30 (br s, 1H, 13-CH_2), 4.01 (dd, 1H, *J*= 10.6, 3.6 Hz, H-16), 2.34 (m, 1H, H-12), 2,20 (m, 2H, H-14, H-15), 2.04 (m, 1H, H-11), 1.93 (m, 2H, H-14, H-15), 1.61 (br d, 2H, *J*= 15 Hz, H-11), 1.53 (s, 3H, 2-Me), 1.08 (s, 3H, 17-Me), 1.07 (s, 3H, 2-Me), 0.62 (s, 3H, 2-Me); **^{13}C NMR (100 MHz, $CDCl_3$):** δ 194.06 (qC, C-10), 188.52 (qC, C-5), 165.83 (qC, C-8), 164.36 (qC, C-6), 145.34 (qC, C-13), 137.02 (CH, C-4), 136.90 (qC, C-4a), 135.79 (qC, C-9a), 111.64 (qC, C-5a), 109.58 (CH_2, 13-CH_2), 109.17 (CH, C-7), 108.49 (CH, C-9), 82.39 (qC, C-10a), 76.66 (qC, C-2), 66.43 (CH, C-16), 59.56 (CH, C-3), 48.07 (CH, C-12), 42.18 (qC, C-17), 37.61 (CH_2, C-14), 36.03 (CH_2, C-11), 35.99 (CH_2, C-15), 28.45 (CH_3, 17-Me), 27.25 (CH_3, 2-Me), 20.38 (CH_3, 2-Me), 16.60 (CH_3, 17-Me); **HR-MS *m*/*z*** 521.0738 (calcd for $C_{25}H_{27}{}^{79}Br^{35}ClO_5$, $[M-H]^-$, 521.0736); **R_T:** 81.5 min.

4-dehydro-4a-dechloro-16-oxonapyradiomycin A80915A (12):
Orange oil (7.40 mg); **UV λ_{max} (nm):** 193.2, 258.6, 314.6; **IR NaCl ν_{max} (cm^{-1}):** 3329.40, 1696.48, 1605.55, 1442.64, 2977.05, 2931.59, 760.67; **^{1}H NMR (400 MHz, $CDCl_3$):** δ 12.36 (s, 6-OH), 7.18 (s, 1H, H-9), 6.86 (d, 1H, 1.6 Hz, H-4), 4.78 (br s, 1H, 13-CH_2), 4.47 (d, 1H, *J*= 1.3 Hz, H-3), 3.86 (br s, 1H, 13-CH_2), 3.73 (dd, 1H, *J*= 11.6, 3.9 Hz, H-16), 2.33 (m, 1H, H-12), 2.24 (br s, 3H, 7-Me), 2,00 (m, 2H, H-14, H-15), 1.90 (m, 1H, H-11), 1.75 (m, 2H, H-14, H-15), 1.61 (br d, 2H, *J*= 15 Hz, H-11), 1.53 (s, 3H, 2-Me), 1.08 (s, 3H, 17-Me), 1.07 (s, 3H, 2-Me), 0.62 (s, 3H, 2-Me); **HR-MS *m*/*z*** 491.23 (calcd for $C_{26}H_{30}{}^{35}Cl_2O_5$, $[M-H]^-$, 491.1398); **R_T:** 30.6 min.

3. In silico environmental toxicity assessment for approved drugs and biocides

Table S1. Toxicity end point predictions for seven Prestwick approved drugs.

	**Approved drugs**						
**Toxicity end points for consensus models**	**S1**[a]	**S2**	**S3**	**S4**	**S5**	**S6**	**S7**
**Fathead minnow 96 hour LC_{50} (mg/L)**	0.26	1.87	0.96	0.22[d]	0.88	0.0117	0.22
***Daphnia magna* 48 hour LC_{50} (mg/L)**	15.80	21.03	8.74	10.35	4.40	0.72	7.73
***Tetrahymena pyriformis* 48 hour IGC_{50} (mg/L)**	3.14	22.03	2.97	14.00[d]	13.49[d]	1.20[d]	1.71

**Oral rat LD_{50} (mg/kg)**	205.85	1127.76	242.69	438.20d	186.97	1.67	396.16
**R phrases, danger symbol, ATE category**	N, R50, 3	N R50, 4	N, R50, 3	N, R50, 4	N, R50, 3	N, R50, 1	N, R50, 4
**Bioconcentration factor**	4.87	3.94	52.94[d]	6.82[d]	61.52	67.81	21.56
**Developmental toxicity**	0.45; DNT[b]	0.75; DT[b]	0.78; DT[b]	1.01; DT[b]	0.87; DT[b]	0.90; DT[b]	0.52; DT[b]
**Ames mutagenicity**	0.17; MN[c]	0.22; MN[c]	0.07; MN[c]	0.16; MN[c]	0.26; MN[c,e]	0.01; MN[c]	0.07; MN[c]

S1 – Bimatoprost, topical medication used for controlling the progression of glaucoma or ocular hypertension, **S2** – Alfuzosin, nonselective alpha-1 adrenergic antagonist used in the therapy of benign prostatic hypertrophy, **S3** – Lovastatin is a fungal metabolite isolated from cultures of *Aspergillus terreus* and is a potent anticholesteremic agent, **S4** – Antimycin A is an antibiotic substance produced by *Streptomyces* species, **S5** – Oxethazaine is an anesthetic, **S6** – Calcipotriene is a synthetic derivative of calcitriol or Vitamin D used for the treatment of moderate plaque psoriasis in adults, **S7** – Latanoprost is a prostaglandin F2alpha analogue and a prostanoid selective FP receptor agonist with an ocular hypertensive effect; [b] DNT - Developmental Non-Toxicant and DT - Developmental Toxicant; [c] Mutagenicity Negative; [d] Predicted by the Nearest Neighbor model, the other models are unable to predict this end point; [e] this compound is in the training set of the mutagenicity model.

Table S2. Toxicity end point predictions for two antifouling approved drugs.

Toxicity end points for consensus models	Ivermectin B1b (S8)	Ivermectin B1a (S9)
**fathead minnow 96 hour LC_{50} (mg/L)**	0.0541	0.00176[a]
***Daphnia magna* 48 hour LC_{50} (mg/L)**	12.91	15.81
***Tetrahymena pyriformis* 48 hour IGC_{50} (mg/L)**	10.77[a]	75.78
**Oral rat LD_{50} (mg/kg)**	29.69	30.31
**R phrases, danger symbol, ATE category**	N, R50, 2	N, R50, 2
**Bioconcentration factor**	1.99	2.20
**Developmental toxicity**	0.44; DNT[b]	0.50; DNT[b]
**Ames mutagenicity**	0.13; MN[c]	0.25; MN[c]

[a] Predicted by the Nearest Neighbor model, the other models are unable to predict this end point; [b] DNT - Developmental Non-Toxicant; [c] Mutagenicity Negative.

Table S3. Aquatic toxicity, environmental fate data and classification of copper and arsenic, experimental data (Tisler and Zagorc-Koncan, 2003).

Toxicity end points	Copper	Arsenic
***Aquatic toxicity Fish Orcorhynchus mykiss* 96 hour LC_{50} (mg/L)**	0.48	15.3
***Aquatic toxicity Daphnia magna* EC_{50}**	0.030	2.5

**48 hour (mg/L)**		
**Aquatic toxicity Alga *Scenedesmus quadricauda* 72 hour IC_{50} (mg/L)**	0.18	34.7
**Chronic toxicity *Daphnia magna* 21 day NOEC (mg/L)**	0.015	1.85
**Acute toxicity[a] Oral rat/rabit LD_{50} (mg/kg)**	140	12
**ATE category**	3	2
**Biodegration**	Irrelevant	Irrelevant
**R phrases, danger symbol**	R50/53, N	R50/53, N

[a] Acros Organics safety data sheet.

References

1. Wu, Z.C.; Li, S.M.; Li, J.; Chen, Y.C.; Saurav, K.; Zhang, Q.B.; Zhang, H.B.; Zhang, W.J.; Zhang, W.M.; Zhang, S., et al. Antibacterial and Cytotoxic New Napyradiomycins from the Marine-Derived Streptomyces sp SCSIO 10428. *Marine Drugs* **2013**, *11*, 2113-2125, doi:10.3390/md11062113.
2. Shiomi, K.; Nakamura, H.; Iinuma, H.; Naganawa, H.; Isshiki, K.; Takeuchi, T.; Umezawa, H. Structures of New Antibiotics Napyradiomycins. *Journal of Antibiotics* **1986**, *39*, 494-501.
3. Motohashi, K.; Sue, M.; Furihata, K.; Ito, S.; Seto, H. Terpenoids produced by actinomycetes: Napyradiomycins from Streptomyces antimycoticus NT17. *Journal of Natural Products* **2008**, *71*, 595-601, doi:10.1021/np070575a.
4. Gomi, S.; Ohuchi, S.; Sasaki, T.; Itoh, J.; Sezaki, M. Studies on New Antibiotics-SF2415 .2. The Structural Elucidation. *Journal of Antibiotics* **1987**, *40*, 740-749, doi:10.7164/antibiotics.40.740.
5. Shiomi, K.; Nakamura, H.; Iinuma, H.; Naganawa, H.; Takeuchi, T.; Umezawa, H.; Iitaka, Y. New Antibiotics Napyradiomycns A2 and B4 and Stereochemistry of Napyradiomycins. *Journal of Antibiotics* **1987**, *40*, 1213-1219.
6. Cheng, Y.B.; Jensen, P.R.; Fenical, W. Cytotoxic and Antimicrobial Napyradiomycins from Two Marine-Derived Streptomyces Strains. *European Journal of Organic Chemistry* **2013**, 3751-3757, doi:10.1002/ejoc.201300349.
7. Fukuda, D.S.; Mynderse, J.S.; Baker, P.J.; Berry, D.M.; Boeck, L.D.; Yao, R.C.; Mertz, F.P.; Nakatsukasa, W.M.; Mabe, J.; Ott, J., et al. A80915, A New Antibiotic Complex Produced by Streptomyces aculeolatus - Discovery, Taxonomy, Fermentation, Isolation, Characterization, and Antibacterial Evaluation. *Journal of Antibiotics* **1990**, *43*, 623-633, doi:10.7164/antibiotics.43.623.
8. Soria-Mercado, I.E.; Prieto-Davo, A.; Jensen, P.R.; Fenical, W. Antibiotic terpenoid chloro-dihydroquinones from a new marine actinomycete. *Journal of Natural Products* **2005**, *68*, 904-910, doi:10.1021/np058011z.
9. Soria-Mercado, I.E.; Jensen, P.R.; Fenical, W.; Kassel, S.; Golen, J. 3,4a-Dichloro-10a-(3-chloro-6-hydroxy-2,2,6-trimethylcyclohexylmethyl)-6 ,8-dihydroxy-2,2,7-trimethyl-3,4,4a,10a-tetrahydro-2H-benzo g chromene-5 ,10-dione. *Acta Crystallographica Section E-Crystallographic Communications* **2004**, *60*, O1627-O1629, doi:10.1107/s1600536804020094.

© 2020 by the authors. Submitted for possible open access publication under the terms and conditions of the Creative Commons Attribution (CC BY) license (http://creativecommons.org/licenses/by/4.0/).
